# Pathogenic mechanisms of amyotrophic lateral sclerosis-linked VAPB P56S mutation in the degeneration of corticospinal motor neurons

**DOI:** 10.20517/and.2025.21

**Published:** 2025-08-03

**Authors:** Xuan Yang, Jiayin Zheng, Xinyu Wang, Huaibin Cai, Jia Yu

**Affiliations:** 1 Basic Research Center, Institute for Geriatrics and Rehabilitation, Beijing Geriatric Hospital, Beijing 100095, China.; 2 Transgenic Section, Laboratory of Neurogenetics, National Institute on Aging, National Institutes of Health, Bethesda, MD 20892, USA.

**Keywords:** Vesicle-associated membrane protein-associated protein B (VAPB), mutation, corticospinal motor neuron (CSMN), neurodegeneration, amyotrophic lateral sclerosis (ALS)

## Abstract

**Aim:**

The endoplasmic reticulum (ER)-localized vesicle-associated membrane protein-associated protein B (VAPB) is implicated in many cellular processes, such as ER-organelle tethering, calcium homeostasis, and unfolded protein response. The P56S missense mutation in VAPB has been associated with familial forms of motor neuron diseases such as typical amyotrophic lateral sclerosis (ALS), atypical ALS, and spinal muscular atrophy. However, it has not been determined how the VAPB P56S mutation induces the degeneration of corticospinal motor neurons (CSMNs) in ALS.

**Methods:**

Using homozygous knock-in (KI) mice expressing P56S VAPB, we investigated the mutation’s pathogenic impacts and underlying mechanisms on the survival and function of CSMNs. We performed a wide variety of assays to examine the behavioral, histological, cellular, and molecular abnormalities of KI mice.

**Results:**

Compared with wild-type controls, KI mice showed the downregulated protein level of mutant VAPB, proteinase K-resistant cytoplasmic inclusions of mutant VAPB in CSMNs, abnormal hyperactivity, impaired motor coordination, neuronal loss of CSMNs, and axonal degeneration of pyramidal and corticospinal tracts. Mechanistic studies revealed that the VAPB P56S mutation rendered the mutant protein destabilized and inclusion-prone in cortical neurons, and the proteasomal degradation played a critical role in modulating mutant VAPB’s protein level and inclusion formation. In addition, the VAPB P56S mutation disrupted ER-mitochondria contacts, impaired VAPB-PTPIP51 interaction and IP3R-VDAC interaction, elevated cytosolic Ca^2+^, activated CaMKII, and increased CRMP2 phosphorylation. Moreover, the VAPB P56S mutation activated the IRE1-XBP1/p38 mitogen-activated protein kinase (MAPK)/ c-Jun N-terminal kinase (JNK) pathway, increased tau hyperphosphorylation, and upregulated p53 expression and phosphorylation.

**Conclusion:**

These findings demonstrate the progressive degeneration of CSMNs induced by VAPB P56S mutation and indicate the involvement of the Ca^2+^-CaMKII-CRMP2 and IRE1-p38 MAPK/JNK-tau/p53 pathways in the pathogenic process.

## INTRODUCTION

The endoplasmic reticulum (ER), an extensive and dynamic network forming membrane contact sites with various intracellular organelles, plays crucial roles in calcium storage and release, lipid synthesis and transfer, protein processing and trafficking, and stress sensing and responding^[1–6]^. ER-organelle membrane contact sites are mainly conferred by the vesicle-associated membrane protein-associated proteins [VAPs, including VAPA, vesicle-associated membrane protein-associated protein B (VAPB), MOSPD1, MOSPD2, and MOSPD3], which are a family of ER-resident type II integral membrane proteins with a conserved Major Sperm Protein (MSP) domain^[7–9]^. VAPs bind diverse interaction partners and tether different organelles to the ER^[10–12]^. Since 2004, the P56S missense mutation in VAPB has been found to cause dominantly inherited motor neuron diseases such as typical amyotrophic lateral sclerosis (ALS), atypical ALS (designated ALS type 8, ALS8), and late-onset spinal muscular atrophy^[13–15]^. In addition to P56S, novel mutations in VAPB (T46I, P56H, A145V, S160Δ, and V234I) have been identified to be associated with ALS^[16–20]^. Dysfunction and loss of motor neurons are reported in ALS patients with the VAPB P56S mutation^[13,21–26]^. However, the pathogenicity of the VAPB P56S mutation in motor neuron degeneration and the potential therapeutic targets remain elusive.

The highly conserved and ubiquitously expressed VAPB and its homolog VAPA are localized throughout the ER sheets and tubules^[27,28]^. VAPB consists of MSP, coiled-coil, and transmembrane domains in the N-terminus, central segment, and C-terminus. The MSP domain, exposed to the cytosol, allows VAPB to interact with the two phenylalanines in an acidic tract (FFAT) motif in diverse target proteins to recruit them to the ER^[9,29]^. The coiled-coil and transmembrane domains allow VAPB and VAPA to undergo homo- and heterodimerization^[9,29]^. The interaction of VAPB with a wide variety of proteins implicates VAPB in many cellular processes, such as ER-organelle tethering, lipid transfer, calcium homeostasis, membrane trafficking, unfolded protein response (UPR), cytoskeleton organization, autophagy, and infection^[9,15,29]^. Unexpectedly, in Caenorhabditis elegans and Drosophila, VAPB’s MSP domain is secreted as an extracellular signaling molecule, indicating VAPB’s non-cell-autonomous function^[30–34]^.

The P56S point mutation causes gross misfolding of the MSP domain and renders the mutant VAPB prone to aggregation^[35,36]^. Accordingly, when overexpressed in transfected mammalian cells and transgenic animal models, P56S VAPB forms intracellular inclusions that dramatically restructure the ER, induce ER stress, and activate the UPR, suggesting a toxic-gain-of-function mechanism in the pathogenicity of P56S VAPB^[13,17,37–55]^. In addition, the intracellular inclusions within cells overexpressing P56S VAPB sequester wild-type VAPB and VAPA, making them unable to function appropriately, implying a dominant-negative effect of P56S VAPB^[38,40,47]^. Besides the aggregation-prone property, the P56S mutation in the MSP domain also compromises the normal function of VAPB by interfering with its interaction with FFAT-containing proteins and thus perturbs the contact/interplay between the ER and organelles^[38,41,56–59]^. Moreover, the protein level of VAPB is reduced in ALS8 patients-derived motor neurons, but the intracellular inclusions of VAPB are not observable^[60,61]^. These studies indicate that a loss-of-function mechanism might also be implicated in VAPB P56S mutation-induced neurodegeneration.

Transgenic and knock-in (KI) rodent models expressing P56S VAPB have been generated to investigate the pathophysiological roles of P56S VAPB. Although all the transgenic mouse lines present P56S VAPB inclusions in motor neurons, only the line generated by us, in which P56S VAPB is highly overexpressed, develops movement disorders and the loss of motor neurons^[51–55]^. Both KI mice and KI rats display motor symptoms and pathological changes in spinal motor neurons (SMNs)^[62,63]^. However, it has not been determined how the P56S mutant VAPB expressed at the physiological level induces the degeneration of corticospinal motor neurons (CSMNs) in ALS.

The current study investigated P56S VAPB’s pathogenic impacts on CSMN’s survival and function using homozygous KI mice. We found that the KI mice displayed the downregulated protein level of mutant VAPB, cytoplasmic inclusions of the mutant VAPB in CSMNs, movement disorders, and progressive degeneration of CSMNs. We further found that the ubiquitin-proteasome system modulated the level and inclusion formation of P56S VAPB in cortical neurons. Moreover, we found that the impairment of ER-mitochondria tethering/interplay, as well as the dysregulation of cytosolic Ca^2+^-calcium/calmodulin-dependent protein kinase type II (CaMKII) and inositol-requiring enzyme 1 (IRE1)-mitogen-activated protein kinase (MAPK) signaling pathways, contributed to the P56S VAPB-induced axonal degeneration and preferential loss of CSMNs.

## METHODS

### Animal maintenance and behavioral test

The *Vapb*^P56S/+^ heterozygous KI mice with the endogenous *Vapb* exon 2 replaced by the P56S mutant *Vapb* exon 2 (JAX, # 028360) were mated to get *Vapb*^+/+^ and *Vapb*^P56S/P56S^ mice [designated as wild-type (WT) and P56S KI mice, respectively]^[62]^. The P56S KI mice and WT controls were also crossed with Thy1-YFP transgenic mice (JAX, #003782) to obtain *Vapb*^P56S/+^;*Thy1-YFP* and *Vapb*^+/+^;*Thy1-YFP* (named as WT/Thy1-YFP) mice, respectively^[64]^. The *Vapb*^P56S/+^;*Thy1-YFP* and P56S KI mice were further bred to produce *Vapb*^P56S/P56S^;*Thy1-YFP* (named as P56S KI/Thy1-YFP) mice. In Thy1-YFP transgenic mice, pyramidal neurons in cortex Layer V are selectively labeled with the yellow fluorescent protein, and their axons in the pyramidal tract and corticospinal tract are brightly fluorescent^[65,66]^. This allows us to examine the axon pathways of CSMNs in the P56S KI/Thy1-YFP mice and gender/age-matched WT/Thy1-YFP controls. Mice were housed, fed, and handled following the guidelines approved by the Institutional Animal Care and Use Committees of Beijing Geriatric Hospital.

Genotyping polymerase chain reaction (PCR) was performed using tail genomic DNA extracted with the One Step Mouse Genotyping Kit (Vazyme). Specific PCR primers (5’-ACAGAGCCATCAGCACACAC-3’ and 5’-AATCTACACCCGAGCAAGTGA-3’ for the WT or P56S *Vapb* gene; 5’-CGGTGGTGCAGATGAACTT-3’ and 5’-ACAGACACACACCCAGGACA-3’ for the *Thy1-YFP* transgene) were used^[62,64]^.

Large cohorts (n ≥ 25 male animals per genotype) of 12-month-old P56S KI mice and littermate WT controls were assessed by behavioral test performers blinded to genotypes. Mouse’s motor functions, including the grip strength of forelimbs or hindlimbs, severity score in the hindlimb clasping test, locomotion and center preference in the open field test, latency to fall in the rotarod test, arm entries in the Y maze, and total distance traveled in the habituation session of the novel object recognition test, were evaluated as we described previously^[53,67,68]^. Mouse’s cognitive functions, including the percentage of alternations in the Y maze and discrimination index and novelty preference in the trial session of the novel object recognition test, were analyzed by the Automated Behavior Analysis Systems (Clever Sys, Inc.)^[67]^. Note that mice exhibiting an object bias score [= (Time Exploring One Particular Object) ÷ (Total Time Exploring Two Identical Objects)] in the pretrial session below 0.20 or above 0.80 were excluded from further experimentation in the novel object recognition test.

### Histology, proteinase K digestion of sections, immunohistochemistry, and stereology

Perfusion fixation of mice, dissection of brains and spinal cords, preparation of 40-μm-thick frozen sections, and immunofluorescent staining with antibodies [Table 1] were performed as we described previously^[53,68]^. At each time point, three to five mice per genotype were used for immunohistochemistry (IHC), and at least 5 coronal or sagittal sections or 20 neurons per mouse were examined.

To determine the stability of proteins in tissues to proteolytic degradation, brain sections were pretreated with 0 or 100 μg/mL proteinase K (PK, Viagen Biotech) in Tris-EDTA (TE) buffer [50 mm Tris-HCl (pH 7.5), 5 mm EDTA] at 37 °C for 10 min, before IHC.

For stereology, serial sagittal brain sections (every 9th section from Lateral 0.00 to 3.72 mm) and coronal spinal cord sections (every 10th section from L1 to L5) were immunostained with COUP-TF-interacting protein 2 (CTIP2) and choline acetyltransferase (CHAT) antibodies, respectively. CSMNs (CTIP2^+^ neurons in the motor cortex layer V) and lumbar SMNs (CHAT^+^ neurons in the spinal cord ventral horn) were counted using an unbiased stereology software, Stereo Investigator 10 (MicroBrightField)^[53,68]^. At each time point, three to five mice per genotype were used for stereology. Counters were blinded to the sample’s genotype.

### Confocal microscopy and image analysis

Fluorescent images of tissues or cells were captured in z-series stack scans using either the conventional mode or the super-resolution Airyscan mode of the Zeiss confocal microscope (LSM 880). Paired images in figures were acquired with equivalent settings and processed identically. The quantitative assessment of images’ fluorescent intensities and area fractions was performed using ImageJ (NIH). The line scan analysis of images was performed with the Zeiss ZEN software.

### RNA extraction and quantitative Reverse Transcription-PCR

The quantitative Reverse Transcription-PCR (qRT-PCR) analysis of mRNA extracted from mouse cortices was performed using the FastPure Tissue Total RNA Isolation and HiScript II One Step qRT-PCR SYBR Green kits (Vazyme). Specific qPCR primers for the *Vapb* gene (5’-GAAGGTGATGGAAGAGTGCAG-3’ and 5’-CCCGAAGTCCGTCTTCTTC-3’) and the mouse *Gapdh* gene (Qiagen, #330001) were used^[62]^.

### Homogenization and fractionation, protein extraction, and western blotting (WB)

The total lysate was prepared from mouse cortices or cells with 1% SDS-supplemented TES buffer [50 mm Tris-HCl (pH 7.5), 2 mm EDTA, 150 mm NaCl] containing protease & phosphatase inhibitors.

The Triton X-100 soluble and insoluble fractions were extracted from mouse cortices as described previously^[38]^. Cortical tissues were first lysed in 1% Triton X-100-supplemented TES buffer containing protease & phosphatase inhibitors by gentle homogenization (10 strokes with the Dounce homogenizer). After 10-min centrifugation at 16,000 × g, the lysate was separated into the supernatant (which was harvested as the Triton X-100 soluble fraction) and the pellet. The pellet was further extracted in 1% SDS-supplemented TES buffer with sonication. After centrifugation at 16,000 × g for 10 min, the supernatant was collected as the Triton X-100 insoluble fraction. The total lysate and Triton X-100 insoluble fraction were subjected to western blotting.

To isolate the mitochondria-associated membranes (MAM) fraction^[69]^, mouse cortices were first gently homogenized with a Dounce homogenizer (12 strokes) in the isolation buffer [225 mm mannitol, 75 mm sucrose, 0.1 mm ethylene glycol tetraacetic acid (EGTA), 30 mm Tris-HCl (pH 7.5), 1× cocktail of protease and phosphatase inhibitors]. The homogenate was centrifuged at 600 × g for 5 min at 4 °C to remove nuclei and unbroken cells. The resulting supernatant was centrifuged at 10,000 × g for 10 min at 4 °C to pellet crude mitochondria. The supernatant was further centrifuged at 100,000 × g for 30 min at 4 °C to remove light membranes from the cytosolic fraction. The crude mitochondria were resuspended in mitochondrial reconstitution buffer [250 mm mannitol, 0.5 mm EGTA, and 5 mm HEPES (pH 7.3)] and layered on top of a 30% Percoll gradient buffer [225mm mannitol, 1 mm EGTA, 5 mm HEPES (pH 7.3), 30% Percoll, 1× Protease and Phosphatase Inhibitor Cocktails]. After centrifugation at 95,000 × g for 30 min, a dense band of purified mitochondria was localized near the bottom of the gradient, and a diffuse white band containing crude MAM was visible above the mitochondrial band. The crude MAM was collected, diluted with mitochondrial reconstitution buffer, and centrifuged at 6,300 × g for 10 min at 4 °C to remove contaminating mitochondria. The resulting supernatant was further centrifuged at 100,000 × g for 1 h at 4 °C. The pellet was harvested, sonicated in TES lysis buffer with 1% SDS and 1× Protease and Phosphatase Inhibitor Cocktails. After centrifugation at 16,000 × g for 10 min, the supernatant was collected as the MAM fraction. MAM and cytosolic fractions were subjected to western blotting.

Proteins of samples were measured for concentration with a Bicinchoninic acid assay kit, electrophoresed in NuPAGE gels, transferred to nitrocellulose membranes, probed with antibodies [Table 1], detected by the LI-COR Odyssey system, and quantified with Image J.

### Cell culture, chemical treatment, MTT assay, plasmid transfection, cytosolic Ca^2+^ measurement, live imaging, immunocytochemistry ICC, and proximity ligation assay

Primary neurons were prepared from neonatal P56S KI pups and littermate WT controls as previously described^[68]^. After digestion with 5 U/mL papain, neurons were dissociated from mouse cortices, spun down, resuspended in N2/B27-supplemented neuronal medium, plated on poly-D-lysine (PDL)-coated plates or coverslips, and maintained in a CO_2_ incubator. The neuronal medium was changed every three or four days.

After digestion with TrypLE Enzyme, fibroblasts were dissociated from mouse dorsal skins, spun down, resuspended in 10% fetal bovine serum-supplemented Dulbecco’s modified Eagle medium (DMEM), plated on bottom dishes for live imaging or coverslips for immunocytochemistry, and maintained in a CO_2_ incubator. The fibroblast medium was changed every three or four days.

To inhibit protein synthesis or proteasomal degradation, cortical neurons on DIV26 were treated with the vehicle [dimethyl sulfoxide (DMSO)], a protein synthesis inhibitor [1 g/mL cycloheximide (CHX), Millipore], or a proteasome inhibitor (0.1 M MG132, Merck) for 48 h. To inhibit JNK, the vehicle (DMSO) or a JNK inhibitor (1 M SP600125, MedChemExpress) was applied to primary cortical neurons twice a week from DIV14 to 28.

Primary cortical neurons in 96-well plates (10^5^ cells per well) were subjected to 3- (4,5)-dimethylthiahiazo (-z-y1)-3,5-di- phenytetrazoliumromide (MTT) assay to assess the survival rate. On DIV7, 14, 21, and 28, 0.5 mg/mL MTT was added to the neuronal medium, allowing viable mitochondrial succinate dehydrogenase to reduce the MTT to generate formazan crystals. Neurons were then switched to 100 L DMSO to solubilize the formazan crystals, and the absorbance at 570 nm was read. At each time point, 28 to 30 wells of neurons per genotype per condition were examined.

Expression plasmids for Sec61β-GFP and Mito-7-mCherry were purchased from Addgene. Cells were transfected with plasmids using the CalPhos Mammalian Transfection Kit (TaKaRa). Between 24 and 72 h post-transfection, transfected cells were switched to the Live Cell Imaging Solution, and the fluorescent signals of Sec61β-GFP and Mito-7-mCherry were time-lapse recorded (30-s intervals) at 37 °C using a confocal microscope.

After a 30-min incubation with 5 M Fluo-4 AM and a 2-minute wash in HBSS, cells were either switched to the Live Cell Imaging Solution for time-lapse recording of Fluo-4 AM fluorescence (10-s intervals), or fixed for immunocytochemistry. Cytosolic Ca^2+^ levels in live or fixed cells were calculated as relative Fluo-4 AM fluorescence.

After 4% PFA fixation, Triton X-100 permeabilization, and proper blocking, cells were immunostained with antibodies [Table 1] for immunocytochemistry (ICC) or proximity ligation assay (PLA). To visualize the proximity ligation assay (PLA) signals, antibody-stained cells were further processed with the Duolink PLA kit (Sigma-Aldrich). At each time point, four to six independent cultures per genotype were used for ICC or PLA, and at least 20 cells per culture were examined.

### Statistical analysis

Data were presented as mean ± standard error of the mean. The unpaired *t* test, one-way analysis of variance (ANOVA) with Tukey’s multiple comparisons test, two-way ANOVA with Sidak’s multiple comparisons test, and Pearson correlation test were conducted with GraphPad Prism 9. Not significant (ns), *P* ≥ 0.05; *P < 0.05; **P < 0.01; ***P < 0.001; ****P < 0.0001.

## RESULTS

### P56S KI mice show the downregulated protein level of mutant VAPB and proteinase K (PK)-resistant cytoplasmic inclusions of mutant VAPB in CSMNs

**Figure 1.**
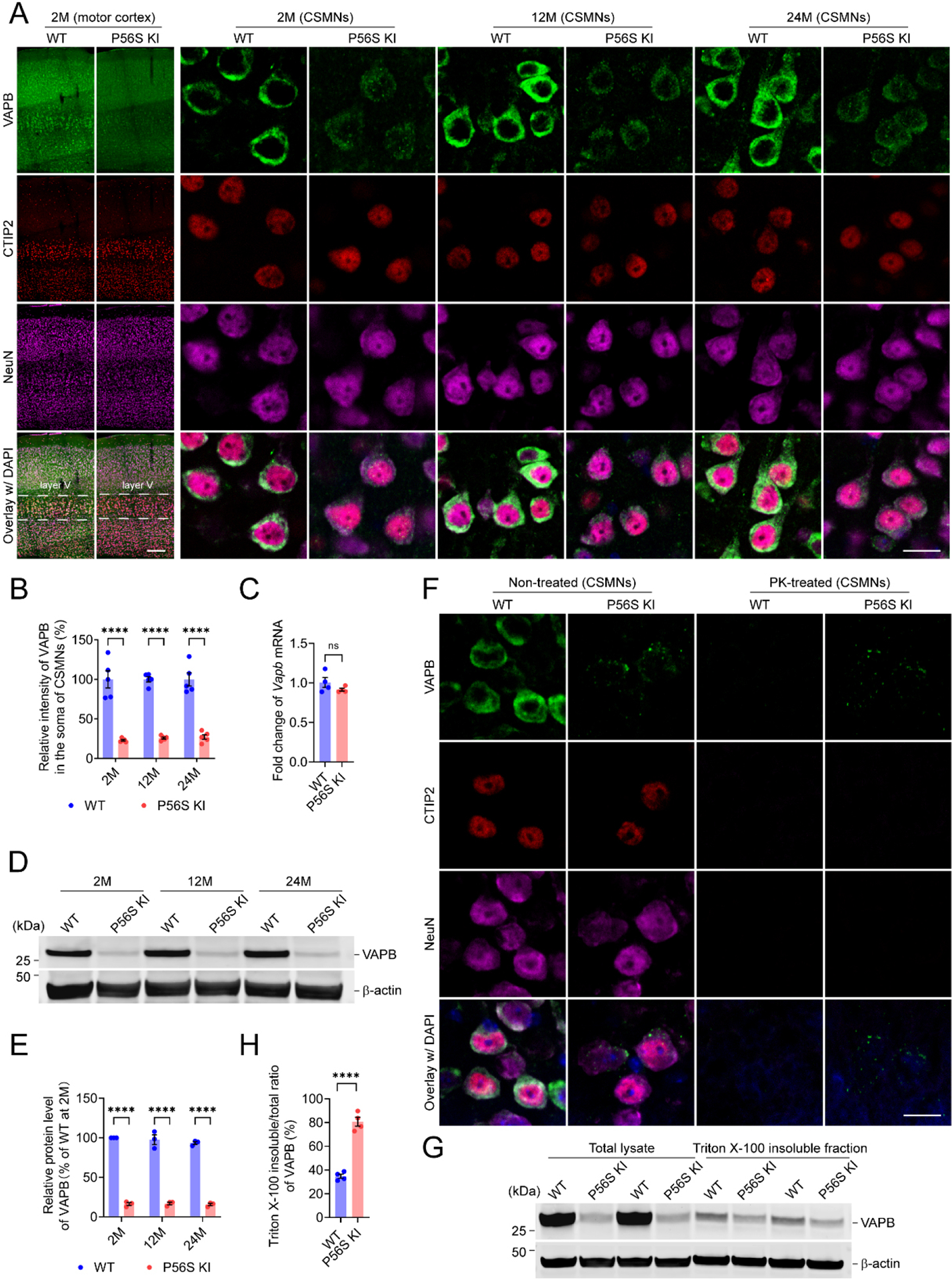
Downregulated protein level of mutant VAPB and PK-resistant cytoplasmic inclusions of mutant VAPB in CSMNs of P56S KI mice. (A and B) Immunostaining of VAPB, CTIP2, and NeuN in the motor cortex of WT and P56S KI mice at 2, 12, and 24 months of age (*n* = 5 at each time point). CSMNs located in the motor cortex layer V were visualized by CTIP2 staining. Note the reduced intensity and cytoplasmic inclusions of mutant VAPB in the CSMNs of P56S KI mice; (C) Quantitative RT-PCR of cortical *Vapb* mRNA of 2-month-old WT and P56S KI mice (*n* = 4); (D and E) Western blotting of VAPB in the cortical total lysate of WT and P56S KI mice at 2, 12, and 24 months of age (*n* = 3 at each time point); (F) Immunostaining of VAPB, CTIP2, and NeuN in the CSMNs of 2-month-old WT and P56S KI mice under non-treated and PK-treated conditions (for each condition, 3 mice per genotype and 5 sections per mouse were examined). Note that no staining of CTIP2, NeuN, or WT VAPB remained after PK digestion, but the PK-resistant cytoplasmic inclusions of P56S VAPB were still visible in P56S KI mice. DAPI (blue) was used to label nuclei; (G and H) Western blotting of VAPB in the total lysate and 1% Triton X-100 insoluble fraction extracted from the cortices of 2-month-old WT and P56S KI mice (*n* = 4). Scale bar: 200 μm (motor cortex) and 20 μm (CSMNs) in (A); 20 μm in (F). Two-way ANOVA: ****P < 0.0001 (2M, 12M, and 24M) in (B); ****P < 0.0001 (2M, 12M, and 24M) in (E); Unpaired *t* test: not significant (ns) *P* = 0.2177 in (C); ****P < 0.0001 in (H). VAPB: Vesicle-associated membrane protein-associated protein B; PK: proteinase K; CSMN: corticospinal motor neuron; KI: knock-in; CTIP2: COUP-TF-interacting protein 2; NeuN: neuronal nuclei; ANOVA: analysis of variance; WT: wild-type.

Since reduced protein levels of VAPB, but no cytoplasmic inclusions, were reported in ALS8 patients’ iPSC-derived motor neurons^[60,61]^, we examined the expression and intracellular distribution pattern of VAPB in the brains and spinal cords of WT and P56S KI mice. Immunohistochemistry revealed a marked reduction in VAPB intensity in the CSMNs of P56S KI mice at 2, 12, and 24 months of age, with approximately 77.0%, 74.1%, and 72.9% decreases, respectively [Figure 1A and B]. In addition to the lower staining intensity, cytoplasmic inclusions of the mutant VAPB were observed in the CSMNs of P56S KI mice [Figure 1A and B]. Consistent with the observations in CSMNs, the downregulated intensity and cytoplasmic inclusions of mutant VAPB were also detected in hippocampal CA2 neurons, striatal neurons, cerebellar Purkinje neurons, and lumbar SMNs of 2-month-old P56S KI mice [Supplementary Figure 1]. The qRT-PCR detected comparable levels of cortical *Vapb* mRNA in 2-month-old WT and P56S KI mice, suggesting the equivalent transcription of *Vapb*^+^ and *Vapb*^P56S^ genes in WT and P56S KI mice, respectively [Figure 1C]. Western blotting of the total lysate extracted from cortical tissues of WT and P56S KI mice confirmed the substantial downregulation of mutant VAPB in P56S KI mice at 2, 12, and 24 months of age, with approximately 83.7%, 80.8%, and 77.9% decreases, respectively [Figure 1D and E].

To examine the stability of cytoplasmic inclusions of P56S VAPB in the CSMNs to proteolytic degradation, we pretreated the motor cortex sections from 2-month-old WT and P56S KI mice with 0 (non-treated condition) or 100 μg/mL proteinase K (PK-treated condition) at 37 °C for 10 min, before immunostaining of VAPB, CTIP2, and NeuN. In the CSMNs of WT mice, no staining of CTIP2, NeuN, or WT VAPB remained after proteinase K (PK) digestion, indicating that PK fully degraded them [Figure 1F]. In the CSMNs of P56S KI mice, no staining of CTIP2 or NeuN was detected following PK digestion, but the PK-resistant cytoplasmic inclusions of P56S VAPB were still visible, implying that cytoplasmic inclusions of mutant VAPB in the CSMNs of P56S KI mice might be in the PK-resistant aggregation state [Figure 1F]. Next, to compare the Triton X-100 insolubility of WT VAPB and P56S VAPB, we immunoblotted the VAPB in the total lysate and 1% Triton X-100 insoluble fraction extracted from the cortices of 2-month-old WT and P56S KI mice. We found that the Triton X-100 insoluble/total ratio of VAPB in P56S KI mice was around 1.3-fold higher than in WT controls, indicating the substantially increased Triton X-100 insolubility of the mutant VAPB [Figure 1G and H].

In a parallel study, we isolated cortices from neonatal WT and P56S KI pups and prepared primary neuronal cultures. Western blotting revealed a gradual increase in VAPB expression in WT cortical neurons from 0 to 28 days in vitro (DIV), while the P56S KI cortical neurons showed a substantial reduction in VAPB expression at each time point compared with WT controls [Supplementary Figure 2A and B]. Immunocytochemistry confirmed the reduced intensity of mutant VAPB in the somata, dendrites, and axons of P56S KI neurons and the cytoplasmic inclusion formation of mutant VAPB [Supplementary Figure 2C and D]. The primary cultures of WT and P56S KI mice may serve as suitable cell models to investigate the pathogenic significance of mutant VAPB in an intensive manner.

### P56S KI mice exhibit movement disorders

**Figure 2.**
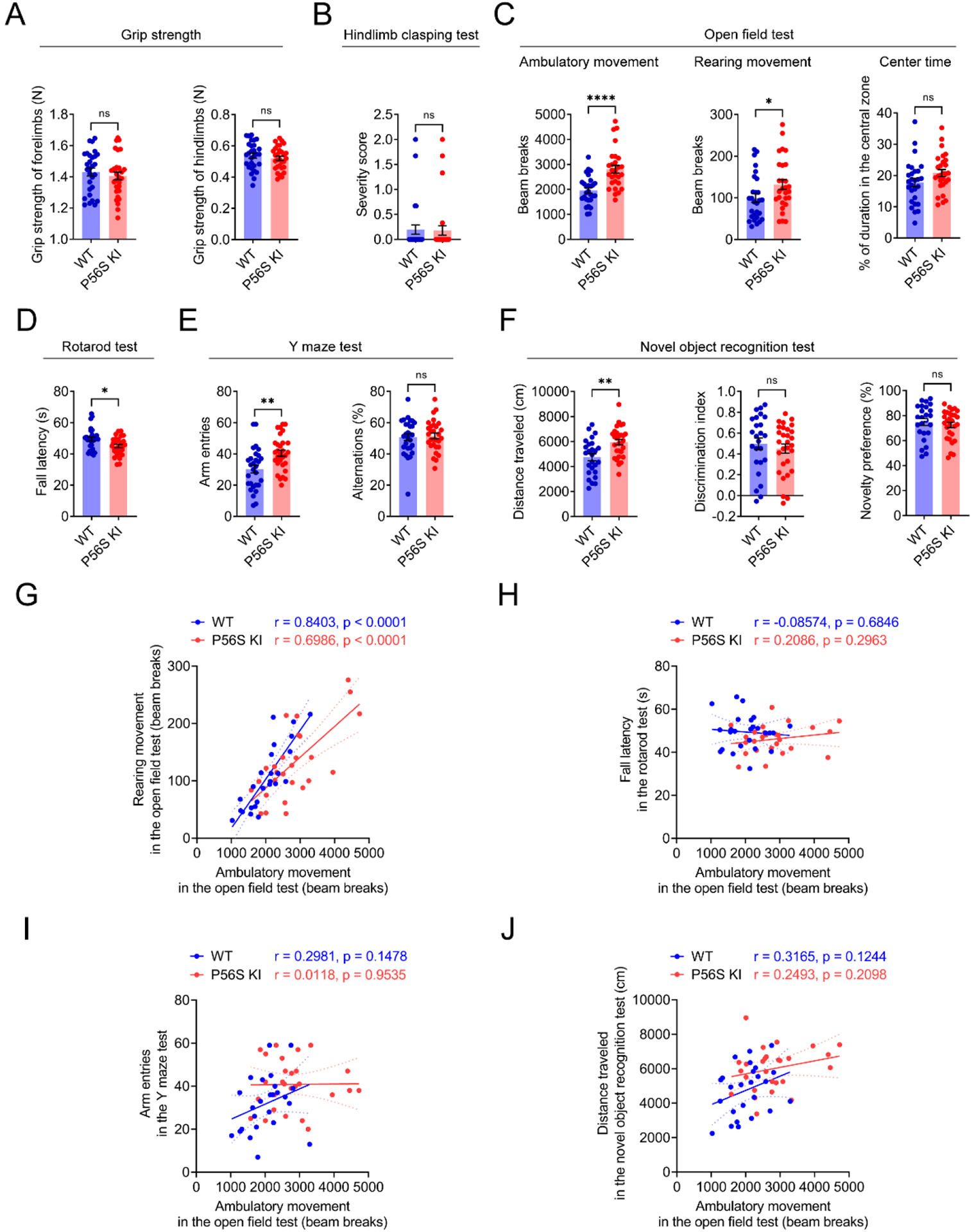
Movement disorders in P56S KI mice. (A-F) At 12 months of age, large cohorts of male WT and P56S KI mice (*n* ≥ 25) were assessed for the grip strength of the forelimbs and hindlimbs (A); the severity score in the hindlimb clasping test (B); the ambulatory movement, rearing movement, and center time in the open field test (C); the latency to fall in the rotarod test (D); the arm entries and percent alternations in the Y maze test (E); and the distance traveled, discrimination index, and novelty preference in the new object recognition test (F); (G-J) Pearson correlation analysis of the ambulatory movement in the open field test with the rearing movement in the open field test (G); the fall latency in the rotarod test (H); the arm entries in the Y maze test (I), and the distance traveled in the novel object recognition test (J). Unpaired *t* test: not significant (ns) *P* = 0.4879 (forelimbs) and ns *P* = 0.4347 (hindlimbs) in (A); ns *P* = 0.8912 in (B); ****P < 0.0001 (ambulatory movement), *P = 0.0451 (rearing movement), and ns *P* = 0.0923 (center time) in (C); *P = 0.0107 in (D); **P = 0.0021 (arm entries) and ns *P* = 0.7743 (percent alternations) in (E); **P = 0.0013 (distance traveled), ns *P* = 0.5154 (discrimination index), and ns *P* = 0.5154 (novelty preference) in (F). KI: Knock-in; WT: wild-type.

As physical and mental decline is reported in ALS patients with VAPB P56S mutation^[13,21,26]^, we monitored large cohorts (*n* ≥ 25) of 12-month-old male WT and P56S KI mice for their motor and cognitive functions. In the grip strength measurement and hindlimb clasping test, P56S KI mice performed similarly to the WT controls, with no apparent sign of weakness [Figure 2A and B]. The open field test revealed that P56S KI mice displayed a similar duration in the center area as WT controls, but exhibited substantially increased ambulatory and rearing movements, indicating the hyperactive phenotype [Figure 2C]. The rotarod test revealed a shorter fall latency in P56S KI mice on the rotating rods [Figure 2D], indicating impaired motor coordination. In the Y maze and novel object recognition tests, no significant difference in cognitive function was found between P56S KI mice and WT controls [Figure 2E and F]. However, significantly increased arm entries and distance traveled were found in P56S KI mice, providing additional evidence for their hyperactive phenotype [Figure 2E and F]. Interestingly, Pearson correlation analysis revealed a strong positive correlation between the ambulatory movement and the rearing movement in the open field test, for both WT (*r* = 0.8403, *P* < 0.0001) and P56S KI (*r* = 0.6986, *P* < 0.0001) mice [Figure 2G]. Nevertheless, the ambulatory movement in the open field test was not correlated with the fall latency in the rotarod test, the arm entries in the Y maze test, or the distance traveled in the novel object recognition test, for either WT or P56S KI mice [Figure 2H–J]. Taken together, our data demonstrate the abnormal hyperactivity and impaired motor coordination in P56S KI mice.

### P56S KI mice display progressive degeneration of CSMNs

**Figure 3.**
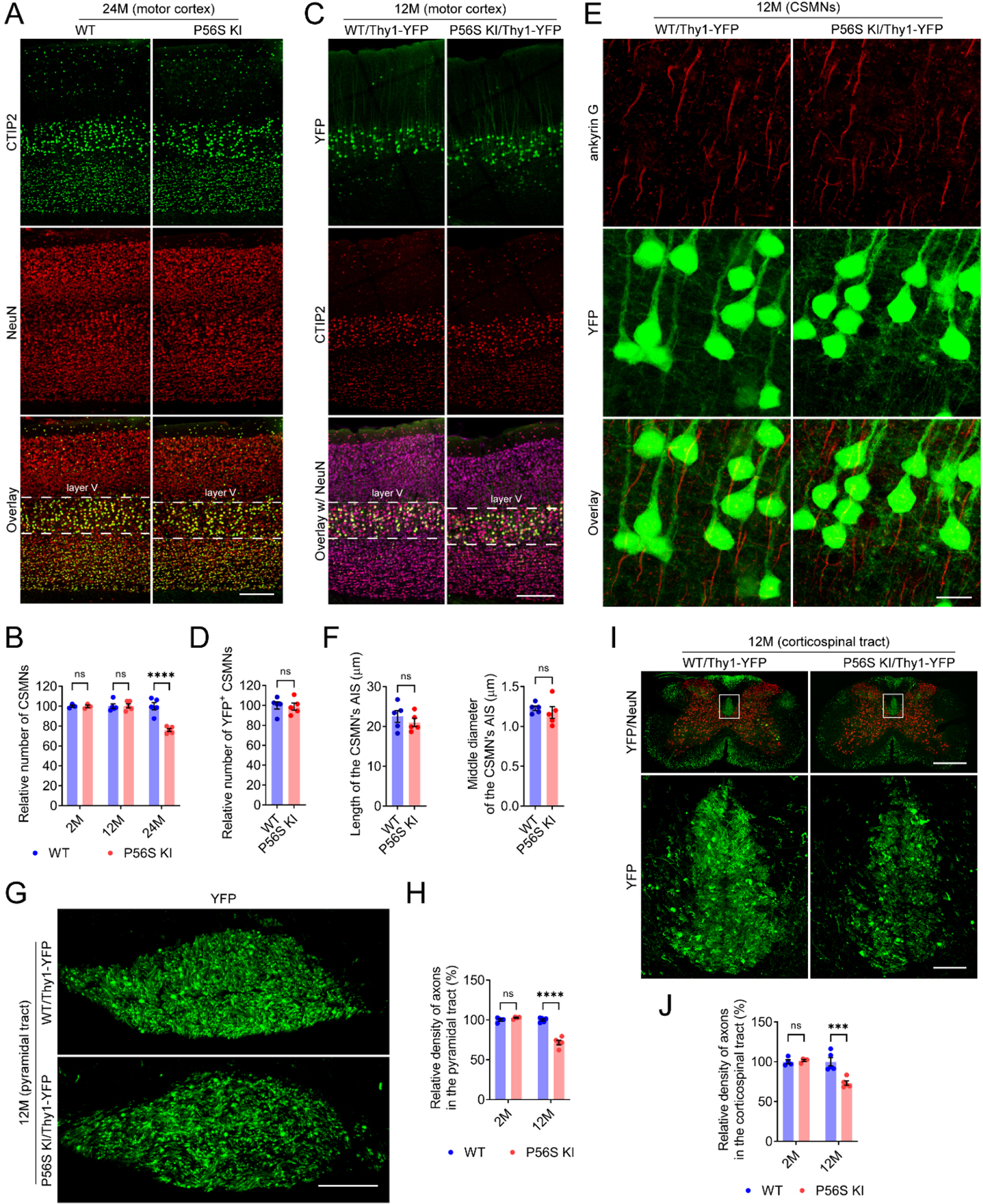
Progressive degeneration of CSMNs in P56S KI mice. (A and B) Immunostaining of CTIP2 and NeuN in the motor cortex of 24-month-old WT and P56S KI mice. CSMNs of 2-, 12-, and 24-month-old WT and P56S KI mice were counted by unbiased stereology (*n* ≥ 3 at each time point); (C and D) Immunostaining of CTIP2 and NeuN (purple) in the motor cortex of 12-month-old WT/Thy1-YFP and P56S KI/Thy1-YFP mice. Unbiased stereological counting of the YFP^+^ CSMNs was performed (*n* = 5); (E and F) Immunostaining of ankyrin G in the motor cortex layer V of 12-month-old WT/Thy1-YFP and P56S KI/Thy1-YFP mice. The length and middle diameter of the CSMN’s AIS were measured (*n* = 5); (G-J) Coronal-section imaging of the YFP-labeled axons in the pyramidal tract at the medulla level (G) and the dorsal corticospinal tract at the cervical spinal cord level (I) of 12-month-old WT/Thy1-YFP and P56S KI/Thy1-YFP mice. The relative density of axons in the pyramidal tract at the medulla level (H) and the dorsal corticospinal tract at the cervical spinal cord level (J) was quantified (*n* ≥ 4 at each time point). Scale bar: 200 μm in (A) and (C); 20 μm in (E);100 μm in (G); 500 μm (upper panel) and 50 μm (lower panel) in (I). Two-way ANOVA: not significant (ns) *P* = 0.999936 (2M), ns *P* = 0.999673 (12M), and ****P < 0.0001 (24M) in (B); ns *P* = 0.6026 (2M) and ****P < 0.0001 (12M) in (H); ns *P* = 0.9439 (2M) and ***P = 0.0001 (12M) in (J). Unpaired *t* test: ns *P* = 0.8251 in (D); ns *P* = 0.4301 (length of AIS) and ns *P* = 0.5211 (middle diameter of AIS) in (F). CSMN: corticospinal motor neuron; KI: knock-in; CTIP2: COUP-TF-interacting protein 2; NeuN: neuronal nuclei; WT: wild-type; AIS: axon initial segment.

Since hyperactivity has been observed in several strains of mouse models with CSMN lesions^[53,70,71]^, we examined the neuropathology of CSMNs and other neuronal types in WT and P56S KI mice. The number of CSMNs in P56S KI mice was similar to that of WT controls at 2 and 12 months of age, but substantially decreased (approximately 23.9%) at 24 months of age [Figure 3A and B]. In contrast, the amounts of hippocampal CA2 neurons, striatal neurons, cerebellar Purkinje neurons, and lumbar spinal motor neurons in aged P56S KI mice showed no apparent decrease [Supplementary Figure 3]. Our results indicate the late-onset preferential loss of CSMNs in P56S KI mice.

In mice, the axons originating from CSMNs descend along the pyramidal tract and corticospinal tract, innervating spinal premotor interneurons and sensory relay neurons, which modulate SMN activity and sensory feedback^[72,73]^. By crossbreeding WT and P56S KI mice with Thy1-YFP transgenic mice, we obtained WT/Thy1-YFP and P56S KI/Thy1-YFP mice, in which CSMNs and their axons in the pyramidal tract and corticospinal tract are brightly fluorescent. Compared with age-matched WT/Thy1-YFP controls, 12-month-old P56S KI/Thy1-YFP mice showed similar numbers of YFP-positive CSMNs and comparable length and diameter of the CSMN’s axon initial segment (AIS), but exhibited significantly reduced axon density in both the pyramidal tract at the medulla level (approximately 28.3%) and the dorsal corticospinal tract at the cervical spinal cord level (approximately 26.7%) [Figure 3C–J]. On the other hand, axon loss in the pyramidal and dorsal corticospinal tracts was not detectable in 2-month-old P56S KI/Thy1-YFP mice [Figure 3G–J]. These data indicate the axonal degeneration of CSMNs before the neuronal loss in P56S KI mice.

### VAPB P56S mutation destabilizes the mutant protein in a proteasome-dependent way

**Figure 4.**
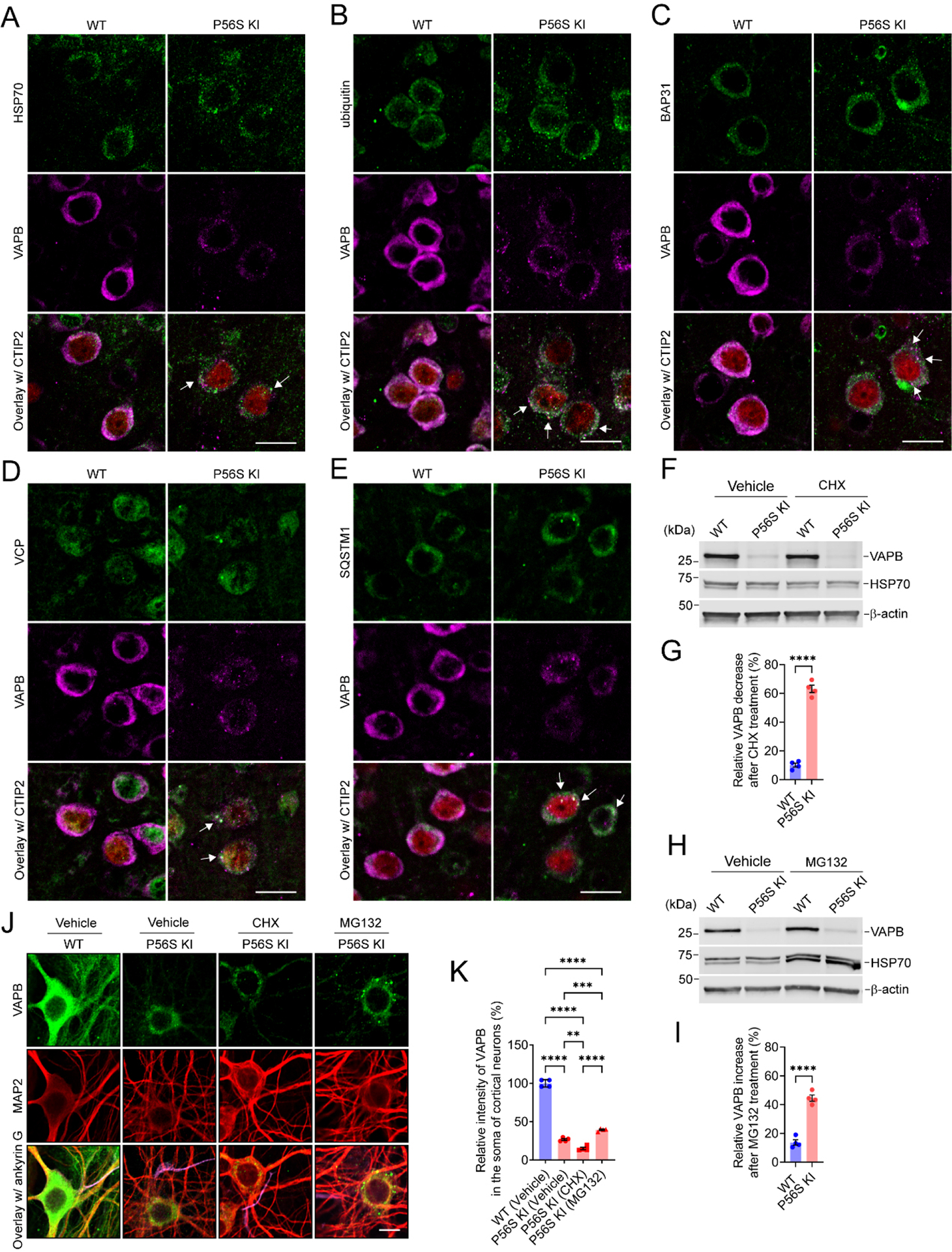
VAPB P56S mutation destabilizes the mutant protein in a proteasome-dependent way. (A-E) Costaining of HSP70 (A); ubiquitin (B); BAP31 (C); VCP (D); or SQSTM1 (E) with VAPB and CTIP2 (red) in the CSMNs of WT and P56S KI mice at 2 months of age. The colocalization of HSP70 (A); ubiquitin (B); BAP31 (C); VCP (D); or SQSTM1 (E) with VAPB inclusions in P56S KI mice was marked with arrows. (F-I) Western blotting of VAPB in DIV28 WT and P56S KI cortical neurons after 48-hour treatment with 1 μg/mL CHX (F) or 0.1 μm MG132 (H). The VAPB decrease after CHX treatment (G) and the VAPB increase after MG132 treatment (I) were quantified (*n* = 4). (J and K) Immunostaining of VAPB, MAP2, and ankyrin G (purple) in DIV28 WT and P56S KI cortical neurons after indicated treatment for 48 h (*n* = 4). Scale bar: 20 μm in (A-E, and J). Unpaired *t* test: ****P < 0.0001 in (G); ****P < 0.0001 in (I); One-way ANOVA: ****P < 0.0001 [P56S KI (vehicle) *vs*. WT], *P* < 0.0001 [P56S KI (CHX) *vs*. WT], *P* < 0.0001 [P56S KI (MG132) *vs*. WT], **P = 0.0016473382 [P56S KI (CHX) *vs*. P56S KI (vehicle)], ***P = 0.0007940139 [P56S KI (CHX) *vs*. P56S KI (vehicle)], and **P = 0.0016473382 [P56S KI (CHX) *vs*. P56S KI (MG132)] in (K). VAPB: Vesicle-associated membrane protein-associated protein B; HSP70: heat shock protein 70; BAP31: B-cell receptor-associated protein 31; VCP: valosin-containing protein; SQSTM1: sequestosome 1; CTIP2: COUP-TF-interacting protein 2; ANOVA: analysis of variance; WT: wild-type.

As demonstrated previously, P56S VAPB inserts post-translationally into ER membranes as efficiently as WT VAPB, but it rapidly clusters to form inclusions within the ER and restructures the ER^[42,45]^. Misfolded and aggregated ER proteins, such as P56S VAPB, are mainly eliminated by ER-associated degradation (ERAD) and ER-phagy^[44–46,54,74]^. Accordingly, immunohistochemistry revealed that P56S VAPB inclusions in the CSMNs of P56S KI mice were markedly enriched in HSP70, ubiquitin, ERAD components (BAP31 and VCP), and autophagy marker SQSTM1 [Figure 4A–E]. Together, the data suggest P56S VAPB inclusions as regions of increased activity in protein folding, ERAD, and autophagy.

To determine whether the VAPB P56S mutation changes the stability of the mutant protein, we treated DIV26 WT and P56S KI cortical neurons with 0 or 1 μg/mL CHX, a protein synthesis inhibitor. After 48-h treatment with 1 μg/mL CHX, the relative VAPB decrease of WT and P56S KI neurons was approximately 10.1% and 63.1%, respectively [Figure 4F and G]. CHX-induced VAPB decrease was exacerbated significantly in P56S KI neurons, as compared with WT neurons, implying the reduced stability of mutant VAPB [Figure 4F and G]. Next, we treated DIV26 WT and P56S KI cortical neurons with 0 or 0.1 μM MG132, a proteasome inhibitor, to investigate whether the reduced stability of P56S VAPB was owing to its proteasomal degradation. After 48-hour treatment with 0.1 μM MG132, the relative VAPB increase in WT and P56S KI neurons was approximately 13.7% and 44.5%, respectively [Figure 4H and I]. MG132-induced VAPB increase was more profound in P56S KI neurons than in WT neurons [Figure 4F and G]. Immunocytochemistry of P56S KI neurons treated with vehicle, CHX, and MG132 confirmed that the CHX treatment resulted in a significant decrease in VAPB intensity, while the MG132 treatment resulted in a substantial increase in VAPB intensity and the formation of much larger P56S VAPB inclusions [Figure 4J and K]. Collectively, our data indicate that the protein level and aggregate size of mutant VAPB highly depend on proteasomal activity.

### VAPB P56S mutation disrupts ER-mitochondria contacts, elevates cytosolic Ca^2+^, activates CaMKII, and increases CRMP2 phosphorylation

**Figure 5.**
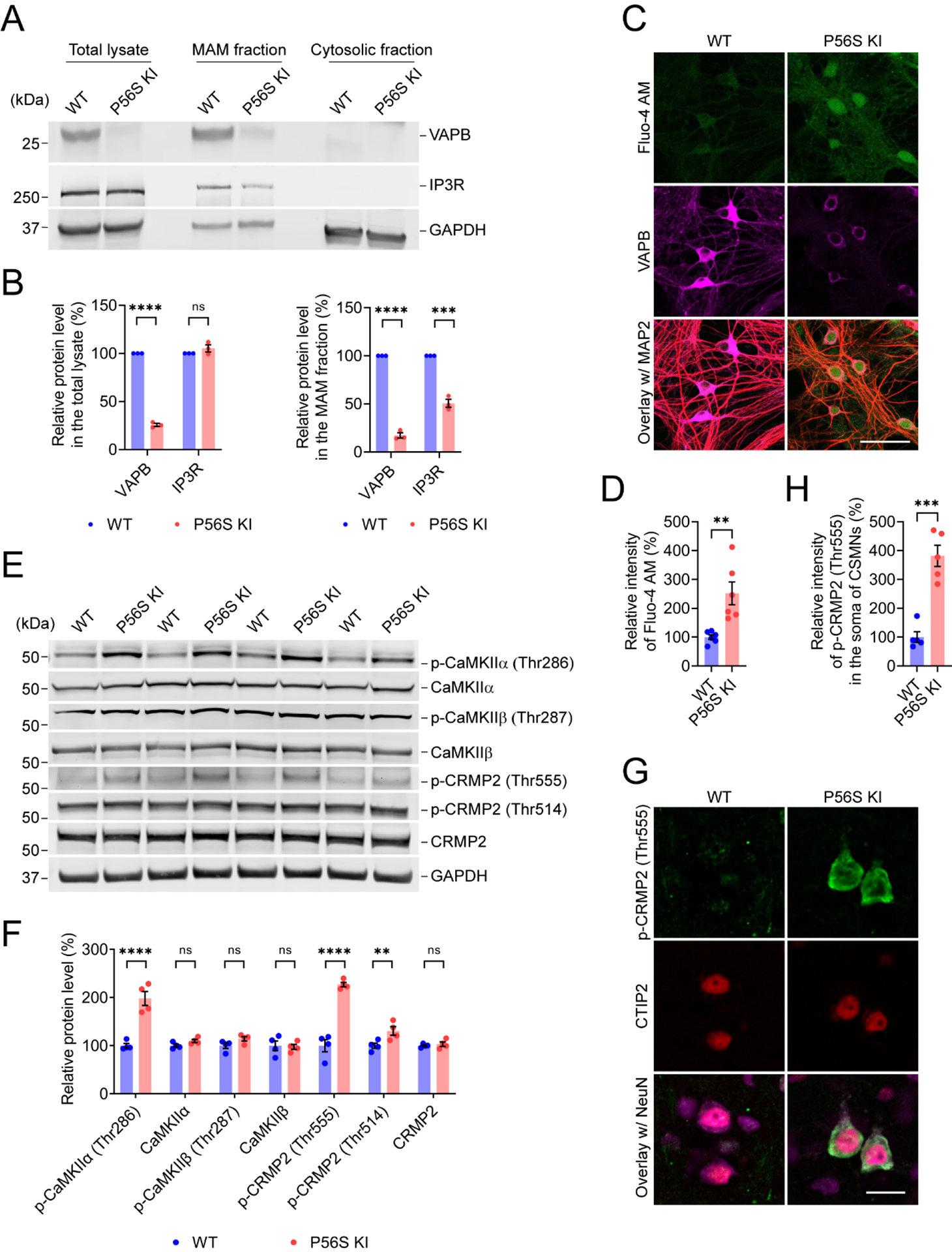
VAPB P56S mutation induces elevation of cytosolic Ca^2+^, activation of CaMKII, and an increase in CRMP2 phosphorylation; (A and B) Western blotting of VAPB and IP3R in the total lysate, MAM fraction, and cytosolic fraction prepared from the cortex of WT and P56S KI mice at 12 months of age (*n* = 3); (C and D) Labeling of Fluo-4 AM in DIV28 WT and P56S KI cortical neurons costained with MAP2 (red) and VAPB (*n* = 6); (E and F) WB of p-CAMKIIα (Thr286), CAMKIIα, p-CAMKIIβ (Thr287), CAMKIIβ, p-CRMP2 (Thr555), p-CRMP2 (Thr514), and CRMP2 in the cortex of WT and P56S KI mice at 12 months of age (*n* = 4); (G and H) Immunostaining of p-CRMP2, CTIP2, and NeuN (purple) in the motor cortex layer V of WT and P56S KI mice at 12 months of age (*n* = 5). Scale bar: 50 μm in (C); 20 μm in (G). Unpaired *t* test: ****P < 0.0001 (VAPB in the total lysate), not significant (ns) *P* = 0.223445 (IP3R in the total lysate), ****P < 0.0001 (VAPB in the MAM fraction), and ***P = 0.000279 (IP3R in the MAM fraction) in (B); **P = 0.0039 in (D); ****P < 0.0001 [p-CaMKIIα (Thr286)], ns *P* = 0.3465 (CaMKIIα), ns *P* = 0.1726 [p-CaMKIIβ (Thr287)], ns *P* = 0.8117 (CaMKIIβ), ****P < 0.0001 [p-CRMP2 (Thr555)], **P = 0.0051 [p-CRMP2 (Thr514)], and ns *P* = 0.7529 (CRMP2) in (F); ***P = 0.000138 in (H). VAPB: Vesicle-associated membrane protein-associated protein B; CaMKII: alcium/calmodulin-dependent protein kinase type II; CRMP2: collapsin response mediator 2; IP3R: inositol 1,4,5-trisphosphate receptor; MAM: mitochondria-associated membranes; WT: wild-type; KI: knock-in; MAP: Thr: threonine; WB: western blotting; MAP2: microtubule-associated protein 2; CaMKIIα: calcium/calmodulin-dependent protein kinase type II subunit alpha; IP3R: inositol 1,4,5-trisphosphate receptor.

VAPB-PTPIP51 (a FFAT motif-containing mitochondrial protein) interaction establishes the ER-mitochondria tethering and facilitates the calcium delivery from ER to mitochondria through IP3R-VDAC interaction, while overexpression of P56S VAPB could disrupt ER-mitochondria contacts and calcium homeostasis^[69,75–78]^. In line with this, immunostaining of BiP (as a marker for ER) and GRP75 (as a marker for mitochondria) revealed the substantially disrupted ER-mitochondria colocalization in P56S KI fibroblasts as compared to the WT cells [Supplementary Figure 4A and B]. Immunocytochemistry also detected the well-organized ER architecture in WT fibroblasts, but expanded perinuclear ER sheets and clustered peripheral ER tubules in P56S KI fibroblasts [Supplementary Figure 4A and B], consistent with the findings that mutant VAPB dramatically restructures the ER^[42,45]^. We further examined mutant VAPB’s effect on ER-mitochondria contact by live imaging of WT and P56S KI fibroblasts co-expressing Sec61β-GFP and Mito-7-mCherry as markers for ER and mitochondria, respectively. In agreement with the immunocytochemistry study, live imaging revealed the noticeably impaired colocalization of ER and mitochondria in P56S KI fibroblasts compared to WT cells [Supplementary Figure 4C and D]. In addition, PLA detected the substantial decrease in VAPB-PTPIP51 interaction and IP3R-VDAC1 interaction in P56S KI fibroblasts compared to WT cells [Supplementary Figure 4E–H]. Western blotting revealed significantly reduced levels of VAPB and IP3R in the MAM fraction prepared from the cortex of P56S KI mice [Figure 5A and B]. Since the VAPB P56S mutation led to the disruption of ER-mitochondria tethering and IP3R-VDAC1 interaction, crucial for calcium homeostasis, we monitored the cytosolic Ca^2+^ levels of WT and P56S KI cells by measuring Fluo-4 AM fluorescence. Compared with cell-type-matched WT controls, P56S KI fibroblasts and cortical neurons exhibited significantly increased intensity of Fluo-4 AM fluorescence, with approximately 153.9% and 151.8% value increases, respectively [Supplementary Figure 4I, J and Figure 5C and D]. These data indicate that the VAPB P56S mutation disrupts ER-mitochondria contacts and elevates cytosolic Ca^2+^.

Ca^2+^ level elevation and calmodulin (CaM) binding acutely activate CaMKII, and the subsequent autophosphorylation of its Thr286 more enduringly activates CaMKII^[79,80]^. Activated CaMKII phosphorylates numerous substrate proteins at serine (Ser) and threonine (Thr), such as CRMP2 at Thr555, with the functional consequences of decreased affinity for tubulin and reduced neurite outgrowth^[81,82]^. Accordingly, 12-month-old P56S KI mice displayed a marked increase in p-CaMKIIα (Thr286) and p-CRMP2 (Thr555) in the cortex [Figure 5E and F] compared to WT controls. Additionally, we also observed a significant increase in p-CRMP2 (Thr514) in the cortex of P56S KI mice, indicating that multiple kinases and phosphatases signaling pathways might be disturbed by the VAPB P56S mutation [Figure 5E and F]. Moreover, immunohistochemistry confirmed the substantial increase (approximately 281.7%) in p-CRMP2 (Thr555) in the CSMNs of 12-month-old P56S KI mice [Figure 5G and H]. Thus, our data demonstrate that the VAPB P56S mutation activates CaMKII and increases CRMP2 phosphorylation, which might result in the axonal degeneration of CSMNs in P56S KI mice.

### VAPB P56S mutation activates IRE1-XBP1/p38 MAPK/JNK pathway, increases tau hyperphosphorylation, and upregulates p53 expression and phosphorylation

**Figure 6.**
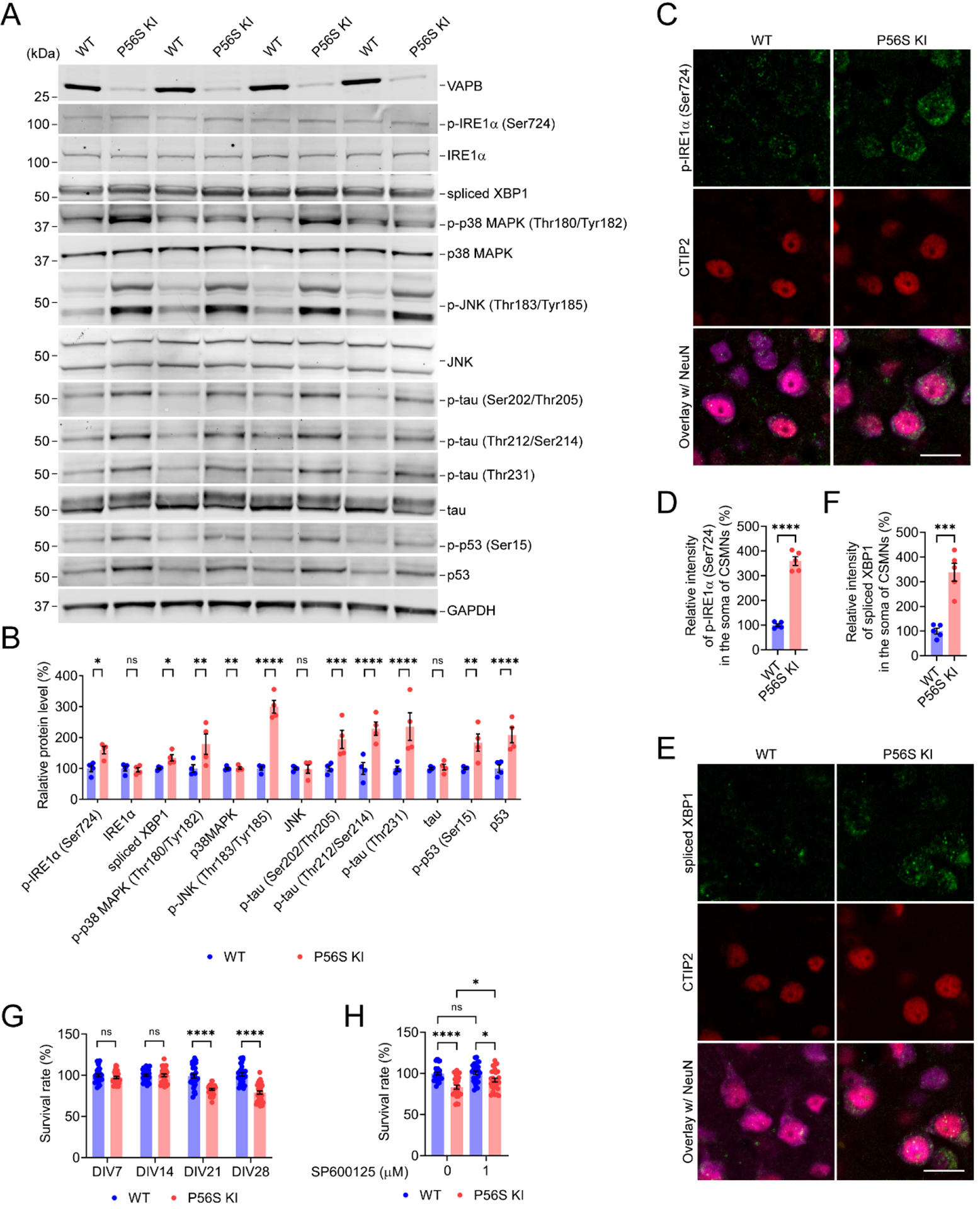
VAPB P56S mutation induces activation of IRE1-XBP1/p38 MAPK/JNK pathway, hyperphosphorylation of tau, and upregulation of total and phosphorylated p53. (A and B) Western blotting of IRE1-XBP1/p38 MAPK/JNK pathway proteins in the cortex of WT and P56S KI mice at 12 months of age (*n* = 4); (C-F) Immunostaining of p-IRE1α (Ser724) (C) or spliced XBP1 (E) in the CSMNs of WT and P56S KI mice at 12 months of age (*n* = 5); (G) The cell viability of cultured WT and P56S KI cortical neurons was measured by MTT assay on DIV7, 14, 21, and 28 (*n* = 30 at each time point); (H) DIV14 WT and P56S KI cortical neurons were treated with 0 or 1 m JNK inhibitor, SP600125, for 14 days and were assayed for cell viability (*n* = 28). Scale bar: 20 μm in (C and E). Unpaired *t* test: *P = 0.0185 [p-IRE1α (Ser724)], not significant (ns) *P* = 0.8588 (IRE1α), * *P* = 0.0173 (spliced XBP1), **P = 0.0020 [p-p38 MAPK (Thr180/Tyr182)], ns *P* = 0.9443 (p38MAPK), ****P < 0.0001 [p-JNK (Thr183/Tyr185)], ns *P* = 0.9267 (JNK), ***P = 0.0003 [p-tau (Ser202/Thr205)], **** *P* < 0.0001 [p-tau (Thr212/Ser214)], ****P < 0.0001 [p-tau (Thr231)], ns *P* = 0.8587 (tau), **P = 0.0011 [p-p53 (Ser15)], and ****P < 0.0001 (p53) in (B); ****P < 0.0001 in (D); ***P = 0.0002 in (F). Two-way ANOVA: ns *P* = 0.747006536694 (DIV7), ns *P* = 0.9999 (DIV14), ****P < 0.0001 (DIV7, DIV21, and DIV28) in (G). One-way ANOVA: ****P < 0.0001 (P56S KI *vs*. WT), ns *P* = 0.9898 (WT SP60025 *vs*. WT), *P = 0.0308 (P56S KI SP60025 *vs*. P56S KI), and *P = 0.0214190311 (P56S KI SP60025 *vs*. WT SP60025) in (H). VAPB: Vesicle-associated membrane protein-associated protein B; IRE1: inositol-requiring enzyme 1; XBP1: X-box binding protein 1; MAPK: mitogen-activated protein kinase; JNK: c-Jun N-terminal kinase; WT: wild-type; KI: knock-in; MTT: 3- (4,5)-dimethylthiahiazo (-z-y1)-3,5-di- phenytetrazoliumromide; ANOVA: analysis of variance; Ser: serine.

In eukaryotic cells, the UPR plays a crucial role in sensing ER stress and maintaining ER homeostasis, but can also direct signaling to programmed cell death if restoration of homeostasis fails^[6]^. Since overexpression of P56S VAPB has been reported to induce the UPR^[17,37,39,40,43,53,62]^, we examined a series of UPR-related proteins in the cortical tissues of 12-month-old WT and P56S KI mice. Western blotting revealed that WT and P56S KI mice displayed comparable levels of PERK, p-PERK (Thr982) (the activated PERK), eIF2α, p-eIF2α (Ser51) (the activated eIF2α), ATF4, CHOP, and ATF6 in the cortex [Supplementary Figure 5A and B]. In addition, immunohistochemistry detected a similar intensity of p-eIF2α (Ser51) in the CSMNs of WT and P56S KI mice [Supplementary Figure 5C and D]. These data indicate that the PERK-eIF2α-ATF4-CHOP and ATF6 branches of the UPR signaling pathway are not apparently activated in the cortex of P56S KI mice. By contrast, compared with WT controls, P56S KI mice showed a significant increase in p-IRE1α (Ser724) (the activated IRE1α), spliced XBP1 (the activated XBP1), p-p38 MAPK (Thr180/Tyr182) (the activated p38 MAPK), and p-JNK (Thr183/Tyr185) (the activated JNK) in the cortex [Figure 6A and B]. Immunohistochemistry confirmed the substantially increased intensity of p-IRE1α (Ser724) and spliced XBP1 in the CSMNs of P56S KI mice compared with WT controls [Figure 6C–F]. Thus, our results demonstrate that the IRE1-XBP1/p38 MAPK/JNK branch of the UPR signaling pathway is significantly activated in the cortex of P56S KI mice.

Spliced XBP1 enhances the transcription of target gene products required to restore proteostasis, exerting a pro-survival effect^[83]^. By contrast, sustained activation of p38 MAPK and JNK leads to tau hyperphosphorylation, as well as the stabilization/phosphorylation of p53, ultimately resulting in the degeneration and apoptosis of neurons^[84–86]^. Accordingly, compared with WT controls, P56S KI mice displayed a marked increase in p-tau (Ser202/Thr205), p-tau (Thr212/Ser214), and p-tau (Thr231) in the cortex [Figure 6A and B]. Moreover, we found significant increases in p-p53 (Ser15) and total p53 in the cortex of P56S KI mice [Figure 6A and B]. These data indicate that the VAPB P56S mutation increases tau hyperphosphorylation and upregulates total and phosphorylated p53, which may contribute to axonal degeneration and CSMN loss in P56S KI mice, respectively. Using the MTT assay, we monitored the cell viability of cultured WT and P56S KI cortical neurons. Compared with WT controls, P56S KI cortical neurons showed no marked change in survival rate on 7DIV and 14DIV but exhibited a substantial neuronal loss starting from 21DIV [Figure 6G]. To test whether inhibiting the MAPK signaling pathway might protect cortical neurons against the VAPB P56S mutation-induced death, we applied SP600125 (a JNK inhibitor). Treatment with 1 μM SP600125 from 14 to 28 DIV significantly mitigated the cell death of P56S KI cortical neurons, suggesting the MAPK pathway may be a potential therapeutic target for neurodegeneration in ALS8 [Figure 6H].

## DISCUSSION

This study explored the pathogenic impacts of the ALS-linked VAPB P56S mutation on CSMNs in P56S KI mice. P56S KI mice showed the downregulated protein level of mutant VAPB and cytoplasmic inclusions of mutant VAPB in various types of neurons. P56S KI mice developed abnormal hyperactivity, impaired motor coordination, and preferential loss of CSMNs. Before the neuronal loss, substantial axonal degeneration of CSMNs in pyramidal and corticospinal tracts was observed in P56S KI mice. We further revealed that the VAPB P56S mutation destabilized the mutant protein and made it inclusion-prone. We also identified the crucial role of the proteasome in modulating the protein level and inclusion formation of mutant VAPB. Moreover, we found that the VAPB P56S mutation disrupted ER-mitochondria contacts, elevated cytosolic Ca^2+^, activated CaMKII, and increased CRMP2 phosphorylation. Meanwhile, we demonstrated that the VAPB P56S mutation activated the IRE1-XBP1/p38 MAPK/JNK pathway, increased tau hyperphosphorylation, and upregulated p53 expression and phosphorylation. Finally, we provided evidence that JNK inhibition mitigated the cell death of primary cortical neurons induced by the VAPB P56S mutation.

Since the initial linkage of the P56S mutation in the human *VAPB* gene with motor neuron diseases, despite of the fact that patients carrying the P56S mutation presented with extremely heterogeneous clinical and pathological phenotypes, several murine models expressing P56S VAPB have been generated, aiming to recapitulate some clinical and pathological features of the patients and to examine the pathophysiological roles and underlying mechanisms of P56S VAPB. Several groups created transgenic mouse lines that moderately overexpressed P56S VAPB under the mouse prion promoter, the mouse Thy1.2 promoter, or the chicken β-actin promoter^[51,52,54,55]^. These lines developed P56S VAPB inclusions in motor neurons, but no overt motor phenotype or motor neuron loss was observed^[51,52,54,55]^. On the other hand, we generated transgenic mouse lines that highly overexpressed P56S VAPB, driven by the Thy1.2 promoter^[53]^. Biochemical, behavioral, and pathological analyses of our P56S VAPB transgenic mice revealed a 7-fold overexpression of mutant VAPB compared to the endogenous VAPB in brain lysate, excessive inclusions of VAPB in motor neurons, hyperactivity, motor coordination impairment, gait abnormalities, selective loss of CmmNs, and compromised function of SMNs^[53]^. Although these transgenic mice recapitulate some clinical and pathological features of patients carrying mutant VAPB, the pathophysiological relevance of observations from the overexpression animal models should always be interpreted cautiously.

To unravel the pathogenic effects of P56S VAPB expressed at physiological levels, as in patients, P56S KI mouse and rat models were created^[62,63]^. P56S KI mice displayed P56S VAPB inclusions in SMNs, motor defects in the inverted grid and rotarod tests (using small cohorts of mice, *n* ≤ 9), and mild denervation of SMNs’ axon terminals at the neuromuscular junctions, without apparent loss of SMNs^[62]^. In comparison, P56S KI rats exhibited subtle changes in paw placement and the loss of SMNs^[63]^. Using the homozygous P56 KI mice and age-matched WT controls, we confirmed the presence of P56S VAPB inclusions in SMNs but no loss of SMNs in KI mice; moreover, we further investigated the pathogenic impacts of the VAPB P56S mutation on CSMNs in the current study. We monitored the behaviors of large cohorts of WT and P56S KI mice (≥ 25 animals per genotype). We found abnormal hyperactivity and impaired motor coordination, but no overt cognitive defects in P56S KI mice. We also revealed preferential loss of CSMNs in P56S KI mice, as well as axonal degeneration in pyramidal and corticospinal tracts before the neuronal loss. The hyperactive phenotype of P56S KI mice has also been documented in several other mouse strains with CSMN lesions^[53,70,71]^. This is consistent with the notion that CSMNs in mice innervate inhibitory spinal neuron types but do not directly innervate SMNs, as is the case in humans^[72,73]^. Our findings in the current study demonstrate that P56S VAPB expressed at the physiological level is sufficient to trigger CSMN degeneration.

The P56S mutation alters the conformation of mutant VAPB, thereby increasing its propensity to oligomerize and aggregate^[35,36]^. Thus, when overexpressed in cells and tissues, P56S VAPB forms intracellular inclusions that restructure the ER and induce the UPR and cell death, suggesting a toxic gain-of-function mechanism in its pathogenicity in ALS^[13,17,37,55]^. On the other hand, the aggregation-prone P56S VAPB disrupts its binding to FFAT motif-containing proteins, resulting in compromised ER-organelle contacts/interplay^[38,41,56–59]^. Moreover, decreased VAPB expression but no VAPB inclusions were detected in ALS8 patients’ iPSCs-derived motor neurons, and VAPB knock-out (KO) zebrafish and mouse models showed motor deficits, indicating that a loss-of-function mechanism might also be implicated in VAPB P56S’s pathogenicity in ALS^[19,60,61]^. Accordingly, the current study revealed the downregulated protein level of mutant VAPB and PK-resistant cytoplasmic inclusions of mutant VAPB in CSMNs of P56S KI mice. Moreover, we demonstrated that the VAPB P56S mutation destabilized the mutant protein and that the proteasome played a critical role in regulating the mutant VAPB’s level and inclusion formation in neurons. We further found that the VAPB P56S mutation led to the disruption of ER-mitochondria contacts, elevation of cytosolic Ca^2+^, activation of CaMKII, and an increase in CRMP2 phosphorylation, which might be instrumental in the axonal degeneration of CSMNs in P56S KI mice. In parallel, we found that the VAPB P56S mutation activated the IRE1-XBP1/p38 MAPK/JNK pathway, increased tau hyperphosphorylation, and upregulated p53 expression and phosphorylation, which might contribute to the axonal degeneration and neuronal loss of CSMNs in P56S KI mice. Interestingly, aberrant activation of the MAPK pathway was also identified in degenerating motor neurons of ALS patients and superoxide dismutase 1 (SOD1) mutant mice, a model of familial ALS^[87–92]^. Taken together, our data suggest that both the loss-of-function mechanism and the toxic gain-of-function mechanism might underlie the CSMN degeneration induced by the VAPB P56S mutation.

There are at least four limitations to the present study. First, VAPB plays crucial roles in many ER-centered cellular functions^[9,15,29]^, but this study only probed the pathogenic influences of the VAPB P56S mutation on cytosolic calcium, UPR, and the downstream signaling pathways. Future studies will be necessary to examine whether P56S KI mice exhibit defects in other ER-centered functions, such as membrane trafficking, lipid transfer, and autophagy^[48,55,62,93–95]^. Second, VAPB regulates ER-mitochondria contacts/interplay, and thus may influence the dynamics, function, and turnover of mitochondria^[96,97]^. However, the current study focused on investigating ER abnormalities induced by mutant VAPB. Further investigation will be required to determine whether the disruption of ER-mitochondria contacts in P56S KI mice leads to mitochondrial dysfunction, which may also contribute to the dysfunction and degeneration of CSMNs. Third, since we used homozygous P56S KI mice in this study, we were unable to examine the dominant-negative effect of P56S VAPB. In the future, we can use heterozygous KI mice to test whether P56S VAPB expressed at the physiological level exerts a dominant-negative effect over WT VAPB. Fourth, consistent with the observation that patients carrying the pathogenic P56S VAPB mutation typically manifest late-onset clinical and pathological phenotypes, the current study revealed that the P56S VAPB mutation led to progressive late-onset, but not immediate early, degeneration of CSMNs in P56S KI mice. This suggests that other genes/proteins, environmental factors, or aging may influence the pathogenic effects of the VAPB P56S mutation on CSMNs, as well as the selective vulnerability of CSMNs to this mutation, broadening the scope of our future research.

In summary, we demonstrate that P56S VAPB expressed at the physiological level exerts profound pathogenic effects on CSMN’s survival and function. Moreover, our findings raise the significance of Ca^2+^-CaMKII-CRMP2 and IRE1-p38 MAPK/JNK-tau/p53 pathways in the axonal degeneration and neuronal loss of CSMNs induced by VAPB P56S mutation.

## Supplementary Material

Supplementary Material

## Figures and Tables

**Table 1.** List of primary antibodies used in this study

Antigen	Source	Catalog#	Application
VAPB	Proteintech	14477-1-AP	IHC, ICC, WB
VAPB (CoraLite Plus 647-conjugated antibody)	Proteintech	CL647-14477	IHC, ICC
VAPB	Proteintech	66191-1-Ig	PLA
CTIP2	Abcam	ab18465	IHC
NeuN	Abcam	ab104224	IHC
CHAT	Millipore	AB144P	IHC
GAPDH	Proteintech	60004-1-Ig	WB
β-actin	Sigma-Aldrich	A1978	WB
ankyrin G	Synaptic system	386004	ICC
MAP2	Abcam	ab92434	ICC
HSP70	Proteintech	10995-1-AP	IHC, WB
ubiquitin	Proteintech	10201-2-AP	IHC
BAP31	Proteintech	11200-1-AP	IHC
VCP	Proteintech	10736-1-AP	IHC
SQSTM1	MBL	PM066	IHC
BiP	Abcam	ab21685	ICC
GRP75	Santa Cruz Biotechnology	sc-133137	ICC
PTPIP51	Proteintech	20641-1-AP	PLA
IP3R	Santa Cruz Biotechnology	sc-377518	PLA
IP3R	Thermo Fisher Scientific	PA1-901	WB
VDAC1	Proteintech	55259-1-AP	PLA
CaMKIIα	Thermo Fisher Scientific	MA1-048	WB
p-CaMKIIα (Thr286)	Thermo Fisher Scientific	MA1-047	WB
CaMKIIβ	Thermo Fisher Scientific	13-9800	WB
p-CaMKII β/γ/δ (Thr287)	Thermo Fisher Scientific	PA5-37833	WB
CRMP2	Cell Signaling Technology	9393	WB
p-CRMP2 (Thr555)	ECM bioscience	CP2251	IHC, WB
p-CRMP2 (Thr514)	Abcam	ab62478	WB
IRE1α	Cell Signaling Technology	3294	WB
p-IRE1α (Ser724)	Abcam	ab48187	IHC, WB
spliced XBP1	Proteintech	24858-1-AP	IHC, WB
p38 MAPK	Cell Signaling Technology	8690	WB
p38 MAPK (Thr180/Tyr182)	Cell Signaling Technology	4511	WB
JNK	Cell Signaling Technology	3708	WB
p-JNK (Thr183/Tyr185)	Cell Signaling Technology	4668	WB
tau	Proteintech	10274-1-AP	WB
p-tau (Ser202/Thr205)	Thermo Fisher Scientific	MN1020	WB
p-tau (Thr212/Ser214)	Thermo Fisher Scientific	MN1060	WB
p-tau (Thr231)	Thermo Fisher Scientific	MN1040	WB
p53	Cell Signaling Technology	2524	WB
p-p53 (Ser15)	Cell Signaling Technology	9286	WB
PERK	Proteintech	20582-1-AP	WB
p-PERK (Thr982)	Abcam	ab192591	WB
eIF2α	Thermo Fisher Scientific	AHO0802	WB
p-eIF2α (Ser51)	Abcam	ab32157	IHC, WB
ATF4	Proteintech	10835-1-AP	WB
CHOP	Proteintech	15204-1-AP	WB
ATF6	Proteintech	24169-1-AP	WB

VAPB: Vesicle-associated membrane protein-associated protein B; IHC: immunohistochemistry; ICC: immunocytochemistry; WB: western blotting; CTIP2: COUP-TF-interacting protein 2; NeuN: neuronal nuclei; CHAT: choline acetyltransferase; GAPDH: glyceraldehyde-3-phosphate dehydrogenase; MAP2: microtubule-associated protein 2; HSP70: heat shock protein 70;BAP31: B-cell receptor-associated protein 31; VCP: valosin-containing protein; SQSTM1: sequestosome 1; BiP: binding immunoglobulin protein; GRP75: 75 kDa glucose-regulated protein; PTPIP51: protein tyrosine phosphatase-interacting protein 51; IP3R: inositol 1,4,5-trisphosphate receptor; VDAC1: voltage-dependent anion-selective channel protein 1; CaMKIIα: calcium/calmodulin-dependent protein kinase type II subunit alpha; p: phosphorylated; CRMP2: collapsin response mediator 2; IRE1α: inositol-requiring enzyme 1α; XBP1: X-box binding protein 1; MAPK: mitogen-activated protein kinase; JNK: c-Jun N-terminal kinase; PERK: protein kinase-like endoplasmic reticulum kinase; eIF2α: eukaryotic translation initiation factor 2α; ATF: activating transcription factor; CHOP: CCAAT-enhancer-binding protein homologous protein; Ser: serine; Thr: threonine.

## Data Availability

Data and materials of this study will be available from the corresponding authors upon reasonable request.

## References

[1] Prinz WA, Toulmay A, Balla T. The functional universe of membrane contact sites. Nat Rev Mol Cell Biol. 2020;21:7–24. 31732717 10.1038/s41580-019-0180-9 PMC10619483

[2] Sun S, Zhao G, Jia M, Stay in touch with the endoplasmic reticulum. Sci China Life Sci. 2024;67:230–57. 38212460 10.1007/s11427-023-2443-9

[3] Wenzel EM, Elfmark LA, Stenmark H, Raiborg C. ER as master regulator of membrane trafficking and organelle function. J Cell Biol. 2022:221. 10.1083/jcb.202205135 PMC9481738 36108241

[4] Domingues N, Pires J, Milosevic I, Raimundo N. Role of lipids in interorganelle communication. Trends Cell Biol. 2025;35:46–58. 38866684 10.1016/j.tcb.2024.04.008 PMC11632148

[5] Parkkinen I, Their A, Asghar MY, Sree S, Jokitalo E, Airavaara M. Pharmacological regulation of endoplasmic reticulum structure and calcium dynamics: importance for neurodegenerative diseases. Pharmacol Rev. 2023;75:959–78. 37127349 10.1124/pharmrev.122.000701

[6] Acosta-Alvear D, Harnoss JM, Walter P, Ashkenazi A. Homeostasis control in health and disease by the unfolded protein response. Nat Rev Mol Cell Biol. 2025;26:193–212. 39501044 10.1038/s41580-024-00794-0

[7] Murphy SE, Levine TP. VAP, a Versatile access point for the endoplasmic reticulum: review and analysis of FFAT-like motifs in the VAPome. Biochim Biophys Acta. 2016;1861:952–61. 26898182 10.1016/j.bbalip.2016.02.009

[8] Neefjes J, Cabukusta B. What the VAP: The expanded VAP family of proteins interacting with FFAT and FFAT-related motifs for interorganellar contact. Contact (Thousand Oaks). 2021;4:25152564211012246. 10.1177/25152564211012246 PMC7610837 34036242

[9] James C, Kehlenbach RH. The interactome of the VAP family of proteins: An overview. Cells. 2021;10:1780. 34359948 10.3390/cells10071780 PMC8306308

[10] Loewen CJ, Levine TP. A highly conserved binding site in vesicle-associated membrane protein-associated protein (VAP) for the FFAT motif of lipid-binding proteins. J Biol Chem. 2005;280:14097–104. 15668246 10.1074/jbc.M500147200

[11] Di Mattia T, Wilhelm LP, Ikhlef S, Identification of MOSPD2, a novel scaffold for endoplasmic reticulum membrane contact sites. EMBO Rep. 2018:19. 10.15252/embr.201745453 PMC6030701 29858488

[12] Cabukusta B, Berlin I, van Elsland DM, Human VAPome analysis reveals MOSPD1 and MOSPD3 as membrane contact site proteins interacting with FFAT-related FFNT motifs. Cell Rep. 2020;33:108475. 33296653 10.1016/j.celrep.2020.108475

[13] Nishimura AL, Mitne-Neto M, Silva HC, A mutation in the vesicle-trafficking protein VAPB causes late-onset spinal muscular atrophy and amyotrophic lateral sclerosis. Am J Hum Genet. 2004;75:822–31. 15372378 10.1086/425287 PMC1182111

[14] Borgese N, Navone F, Nukina N, Yamanaka T. Mutant VAPB: Culprit or innocent bystander of amyotrophic lateral sclerosis? Contact (Thousand Oaks). 2021;4:25152564211022515. 10.1177/25152564211022515 PMC10243577 37366377

[15] Kors S, Costello JL, Schrader M. VAP proteins - from organelle tethers to pathogenic host interactors and their role in neuronal disease. Front Cell Dev Biol. 2022;10:895856. 35756994 10.3389/fcell.2022.895856 PMC9213790

[16] Landers JE, Leclerc AL, Shi L, New VAPB deletion variant and exclusion of VAPB mutations in familial ALS. Neurology. 2008;70:1179–85. 18322265 10.1212/01.wnl.0000289760.85237.4e

[17] Chen HJ, Anagnostou G, Chai A, Characterization of the properties of a novel mutation in VAPB in familial amyotrophic lateral sclerosis. J Biol Chem. 2010;285:40266–81. 20940299 10.1074/jbc.M110.161398 PMC3001007

[18] van Blitterswijk M, van Es MA, Koppers M, VAPB and C9orf72 mutations in 1 familial amyotrophic lateral sclerosis patient. Neurobiol Aging. 2012;33:2950.e1–4. 10.1016/j.neurobiolaging.2012.07.004 22878164

[19] Kabashi E, El Oussini H, Bercier V, Investigating the contribution of VAPB/ALS8 loss of function in amyotrophic lateral sclerosis. Hum Mol Genet. 2013;22:2350–60. 23446633 10.1093/hmg/ddt080

[20] Sun YM, Dong Y, Wang J, Lu JH, Chen Y, Wu JJ. A novel mutation of VAPB in one Chinese familial amyotrophic lateral sclerosis pedigree and its clinical characteristics. J Neurol. 2017;264:2387–93. 28993872 10.1007/s00415-017-8628-3

[21] Nishimura AL, Mitne-Neto M, Silva HC, Oliveira JR, Vainzof M, Zatz M. A novel locus for late onset amyotrophic lateral sclerosis/motor neurone disease variant at 20q13. J Med Genet. 2004;41:315–20. 15060112 10.1136/jmg.2003.013029 PMC1735732

[22] Marques VD, Barreira AA, Davis MB, Expanding the phenotypes of the Pro56Ser VAPB mutation: proximal SMA with dysautonomia. Muscle Nerve. 2006;34:731–9. 16967488 10.1002/mus.20657

[23] Kosac V, Freitas MR, Prado FM, Nascimento OJ, Bittar C. Familial adult spinal muscular atrophy associated with the VAPB gene: report of 42 cases in Brazil. Arq Neuropsiquiatr. 2013;71:788–90. 24212516 10.1590/0004-282X20130123

[24] Di L, Chen H, Da Y, Wang S, Shen XM. Atypical familial amyotrophic lateral sclerosis with initial symptoms of pain or tremor in a Chinese family harboring VAPB-P56S mutation. J Neurol. 2016;263:263–8. 26566915 10.1007/s00415-015-7965-3

[25] Guo X, Gang Q, Meng L, Peripheral nerve pathology in VAPB-associated amyotrophic lateral sclerosis with dysautonomia in a Chinese family. Clin Neuropathol. 2020;39:282–7. 32383641 10.5414/NP301281

[26] Leoni TB, Rezende TJR, Peluzzo TM, Structural brain and spinal cord damage in symptomatic and pre-symptomatic VAPB-related ALS. J Neurol Sci. 2022;434:120126. 35007920 10.1016/j.jns.2021.120126

[27] Lev S, Ben Halevy D, Peretti D, Dahan N. The VAP protein family: from cellular functions to motor neuron disease. Trends Cell Biol. 2008;18:282–90. 18468439 10.1016/j.tcb.2008.03.006

[28] Dudás EF, Huynen MA, Lesk AM, Pastore A. Invisible leashes: the tethering VAPs from infectious diseases to neurodegeneration. J Biol Chem. 2021;296:100421. 33609524 10.1016/j.jbc.2021.100421 PMC8005810

[29] Borgese N, Iacomino N, Colombo SF, Navone F. The link between VAPB loss of function and amyotrophic lateral sclerosis. Cells. 2021;10:1865. 34440634 10.3390/cells10081865 PMC8392409

[30] Tsuda H, Han SM, Yang Y, The amyotrophic lateral sclerosis 8 protein VAPB is cleaved, secreted, and acts as a ligand for Eph receptors. Cell. 2008;133:963–77. 18555774 10.1016/j.cell.2008.04.039 PMC2494862

[31] Han SM, El Oussini H, Scekic-Zahirovic J, VAPB/ALS8 MSP ligands regulate striated muscle energy metabolism critical for adult survival in caenorhabditis elegans. PLoS Genet. 2013;9:e1003738. 24039594 10.1371/journal.pgen.1003738 PMC3764199

[32] Han SM, Tsuda H, Yang Y, Vibbert J, Cottee P, Lee SJ, Winek J, Haueter C, Bellen HJ, Miller MA. Secreted VAPB/ALS8 major sperm protein domains modulate mitochondrial localization and morphology via growth cone guidance receptors. Dev Cell. 2012; 22(2):348–362. 22264801 10.1016/j.devcel.2011.12.009 PMC3298687

[33] Schultz J, Lee SJ, Cole T, The secreted MSP domain of C. elegans VAPB homolog VPR-1 patterns the adult striated muscle mitochondrial reticulum via SMN-1. Development. 2017;144:2175–86. 28634272 10.1242/dev.152025 PMC5482996

[34] Kamemura K, Chihara T. Multiple functions of the ER-resident VAP and its extracellular role in neural development and disease. J Biochem. 2019;165:391–400. 30726905 10.1093/jb/mvz011

[35] Shi J, Lua S, Tong JS, Song J. Elimination of the native structure and solubility of the hVAPB MSP domain by the Pro56Ser mutation that causes amyotrophic lateral sclerosis. Biochemistry. 2010;49:3887–97. 20377183 10.1021/bi902057a

[36] Song J Transforming Cytosolic Proteins into “insoluble” and membrane-toxic forms triggering diseases/aging by genetic, pathological or environmental factors. Protein Pept Lett. 2017;24:294–306. 28190398 10.2174/0929866524666170209154001

[37] Kanekura K, Nishimoto I, Aiso S, Matsuoka M. Characterization of amyotrophic lateral sclerosis-linked P56S mutation of vesicle-associated membrane protein-associated protein B (VAPB/ALS8). J Biol Chem. 2006;281:30223–33. 16891305 10.1074/jbc.M605049200

[38] Teuling E, Ahmed S, Haasdijk E, Motor neuron disease-associated mutant vesicle-associated membrane protein-associated protein (VAP) B recruits wild-type VAPs into endoplasmic reticulum-derived tubular aggregates. J Neurosci. 2007;27:9801–15. 17804640 10.1523/JNEUROSCI.2661-07.2007 PMC6672975

[39] Gkogkas C, Middleton S, Kremer AM, VAPB interacts with and modulates the activity of ATF6. Hum Mol Genet. 2008;17:1517–26. 18263603 10.1093/hmg/ddn040

[40] Suzuki H, Kanekura K, Levine TP, ALS-linked P56S-VAPB, an aggregated loss-of-function mutant of VAPB, predisposes motor neurons to ER stress-related death by inducing aggregation of co-expressed wild-type VAPB. J Neurochem. 2009;108:973–85. 19183264 10.1111/j.1471-4159.2008.05857.x

[41] Kim S, Leal SS, Ben Halevy D, Gomes CM, Lev S. Structural requirements for VAP-B oligomerization and their implication in amyotrophic lateral sclerosis-associated VAP-B(P56S) neurotoxicity. J Biol Chem. 2010;285:13839–49. 20207736 10.1074/jbc.M109.097345 PMC2859547

[42] Fasana E, Fossati M, Ruggiano A, A VAPB mutant linked to amyotrophic lateral sclerosis generates a novel form of organized smooth endoplasmic reticulum. FASEB J. 2010;24:1419–30. 20008544 10.1096/fj.09-147850

[43] Langou K, Moumen A, Pellegrino C, AAV-mediated expression of wild-type and ALS-linked mutant VAPB selectively triggers death of motoneurons through a Ca^2+^-dependent ER-associated pathway. J Neurochem. 2010;114:795–809. 20477942 10.1111/j.1471-4159.2010.06806.x

[44] Moumen A, Virard I, Raoul C. Accumulation of wildtype and ALS-linked mutated VAPB impairs activity of the proteasome. PLoS One. 2011;6:e26066. 21998752 10.1371/journal.pone.0026066 PMC3187839

[45] Papiani G, Ruggiano A, Fossati M, Restructured endoplasmic reticulum generated by mutant amyotrophic lateral sclerosis-linked VAPB is cleared by the proteasome. J Cell Sci. 2012;125:3601–11. 22611258 10.1242/jcs.102137

[46] Genevini P, Papiani G, Ruggiano A, Cantoni L, Navone F, Borgese N. Amyotrophic lateral sclerosis-linked mutant VAPB inclusions do not interfere with protein degradation pathways or intracellular transport in a cultured cell model. PLoS One. 2014;9:e113416. 25409455 10.1371/journal.pone.0113416 PMC4237408

[47] Ratnaparkhi A, Lawless GM, Schweizer FE, Golshani P, Jackson GR. A drosophila model of ALS: human ALS-associated mutation in VAP33A suggests a dominant negative mechanism. PLoS One. 2008;3:e2334. 18523548 10.1371/journal.pone.0002334 PMC2390852

[48] Forrest S, Chai A, Sanhueza M, Increased levels of phosphoinositides cause neurodegeneration in a drosophila model of amyotrophic lateral sclerosis. Hum Mol Genet. 2013;22:2689–704. 23492670 10.1093/hmg/ddt118 PMC3674808

[49] Chaplot K, Pimpale L, Ramalingam B, Deivasigamani S, Kamat SS, Ratnaparkhi GS. SOD1 activity threshold and TOR signalling modulate VAP(P58S) aggregation via reactive oxygen species-induced proteasomal degradation in a drosophila model of amyotrophic lateral sclerosis. Dis Model Mech. 2019;12:dmm033803. 10.1242/dmm.033803 PMC6398501 30635270

[50] Thulasidharan A, Garg L, Tendulkar S, Ratnaparkhi GS. Age-dependent dynamics of neuronal VAPB(ALS) inclusions in the adult brain. Neurobiol Dis. 2024;196:106517. 38679111 10.1016/j.nbd.2024.106517

[51] Tudor EL, Galtrey CM, Perkinton MS, Amyotrophic lateral sclerosis mutant vesicle-associated membrane protein-associated protein-B transgenic mice develop TAR-DNA-binding protein-43 pathology. Neuroscience. 2010;167:774–85. 20188146 10.1016/j.neuroscience.2010.02.035

[52] Qiu L, Qiao T, Beers M, Widespread aggregation of mutant VAPB associated with ALS does not cause motor neuron degeneration or modulate mutant SOD1 aggregation and toxicity in mice. Mol Neurodegener. 2013;8:1. 23281774 10.1186/1750-1326-8-1 PMC3538568

[53] Aliaga L, Lai C, Yu J, Amyotrophic lateral sclerosis-related VAPB P56S mutation differentially affects the function and survival of corticospinal and spinal motor neurons. Hum Mol Genet. 2013;22:4293–305. 23771029 10.1093/hmg/ddt279 PMC3792689

[54] Kuijpers M, van Dis V, Haasdijk ED, Amyotrophic lateral sclerosis (ALS)-associated VAPB-P56S inclusions represent an ER quality control compartment. Acta Neuropathol Commun. 2013;1:24. 24252306 10.1186/2051-5960-1-24 PMC3893532

[55] Tripathi P, Guo H, Dreser A, Pathomechanisms of ALS8: altered autophagy and defective RNA binding protein (RBP) homeostasis due to the VAPB P56S mutation. Cell Death Dis. 2021;12:466. 33972508 10.1038/s41419-021-03710-y PMC8110809

[56] Moustaqim-Barrette A, Lin YQ, Pradhan S, Neely GG, Bellen HJ, Tsuda H. The amyotrophic lateral sclerosis 8 protein, VAP, is required for ER protein quality control. Hum Mol Genet. 2014;23:1975–89. 24271015 10.1093/hmg/ddt594 PMC3959812

[57] Yamanaka T, Nishiyama R, Shimogori T, Nukina N. Proteomics-based approach identifies altered ER domain properties by ALS-linked VAPB mutation. Sci Rep. 2020;10:7610. 32376919 10.1038/s41598-020-64517-z PMC7203144

[58] James C, Lenz C, Urlaub H, Kehlenbach RH. Sequestosome 1 Is part of the interaction network of VAPB. Int J Mol Sci. 2021;22:13271. 34948065 10.3390/ijms222413271 PMC8707790

[59] Gao XK, Sheng ZK, Lu YH, VAPB-mediated ER-targeting stabilizes IRS-1 signalosomes to regulate insulin/IGF signaling. Cell Discov. 2023;9:83. 37528084 10.1038/s41421-023-00576-6 PMC10394085

[60] Mitne-Neto M, Machado-Costa M, Marchetto MC, Downregulation of VAPB expression in motor neurons derived from induced pluripotent stem cells of ALS8 patients. Hum Mol Genet. 2011;20:3642–52. 21685205 10.1093/hmg/ddr284 PMC3159551

[61] Oliveira D, Morales-Vicente DA, Amaral MS, Different gene expression profiles in iPSC-derived motor neurons from ALS8 patients with variable clinical courses suggest mitigating pathways for neurodegeneration. Hum Mol Genet. 2020;29:1465–75. 32280986 10.1093/hmg/ddaa069

[62] Larroquette F, Seto L, Gaub PL, Vapb/Amyotrophic lateral sclerosis 8 knock-in mice display slowly progressive motor behavior defects accompanying ER stress and autophagic response. Hum Mol Genet. 2015;24:6515–29. 26362257 10.1093/hmg/ddv360 PMC4614709

[63] Murage B, Tan H, Mashimo T, Jackson M, Skehel PA. Spinal cord neurone loss and foot placement changes in a rat knock-in model of amyotrophic lateral sclerosis Type 8. Brain Commun. 2024;6:fcae184. 38846532 10.1093/braincomms/fcae184 PMC11154649

[64] Feng G, Mellor RH, Bernstein M, Imaging neuronal subsets in transgenic mice expressing multiple spectral variants of GFP. Neuron. 2000;28:41–51. 11086982 10.1016/s0896-6273(00)00084-2

[65] Porrero C, Rubio-Garrido P, Avendaño C, Clascá F. Mapping of fluorescent protein-expressing neurons and axon pathways in adult and developing Thy1-eYFP-H transgenic mice. Brain Res. 2010;1345:59–72. 20510892 10.1016/j.brainres.2010.05.061

[66] Ziogas NK, Koliatsos VE. Primary Traumatic axonopathy in mice subjected to impact acceleration: a reappraisal of pathology and mechanisms with high-resolution anatomical methods. J Neurosci. 2018;38:4031–47. 29567804 10.1523/JNEUROSCI.2343-17.2018 PMC6705930

[67] Miedel CJ, Patton JM, Miedel AN, Miedel ES, Levenson JM. Assessment of spontaneous alternation, novel object recognition and limb clasping in transgenic mouse models of amyloid-β and tau neuropathology. J Vis Exp. 2017. 10.3791/55523 PMC5608159 28605382

[68] Yu J, Lai C, Shim H, Genetic ablation of dynactin p150(Glued) in postnatal neurons causes preferential degeneration of spinal motor neurons in aged mice. Mol Neurodegener. 2018;13:10. 29490687 10.1186/s13024-018-0242-z PMC5831668

[69] De Vos KJ, Mórotz GM, Stoica R, VAPB interacts with the mitochondrial protein PTPIP51 to regulate calcium homeostasis. Hum Mol Genet. 2012;21:1299–311. 22131369 10.1093/hmg/ddr559 PMC3284118

[70] Chen B, Schaevitz LR, McConnell SK. Fezl regulates the differentiation and axon targeting of layer 5 subcortical projection neurons in cerebral cortex. Proc Natl Acad Sci U S A. 2005;102:17184–9. 16284245 10.1073/pnas.0508732102 PMC1282569

[71] Han Q, Cao C, Ding Y, Plasticity of motor network and function in the absence of corticospinal projection. Exp Neurol. 2015;267:194–208. 25792481 10.1016/j.expneurol.2015.03.008

[72] Carmona LM, Thomas ED, Smith K, Tasic B, Costa RM, Nelson A. Topographical and cell type-specific connectivity of rostral and caudal forelimb corticospinal neuron populations. Cell Rep. 2024;43:113993. 38551963 10.1016/j.celrep.2024.113993 PMC11100358

[73] Ueno M, Nakamura Y, Li J, Corticospinal circuits from the sensory and motor cortices differentially regulate skilled movements through distinct spinal interneurons. Cell Rep. 2018;23:1286–1300.e7. 29719245 10.1016/j.celrep.2018.03.137 PMC6608728

[74] Wu SA, Li ZJ, Qi L. Endoplasmic reticulum (ER) protein degradation by ER-associated degradation and ER-phagy. Trends Cell Biol. 2025;35:576–91. 39909774 10.1016/j.tcb.2025.01.002 PMC12227305

[75] Mórotz GM, De Vos KJ, Vagnoni A, Ackerley S, Shaw CE, Miller CC. Amyotrophic lateral sclerosis-associated mutant VAPBP56S perturbs calcium homeostasis to disrupt axonal transport of mitochondria. Hum Mol Genet. 2012;21:1979–88. 22258555 10.1093/hmg/dds011 PMC3315205

[76] Stoica R, De Vos KJ, Paillusson S, ER-mitochondria associations are regulated by the VAPB-PTPIP51 interaction and are disrupted by ALS/FTD-associated TDP-43. Nat Commun. 2014;5:3996. 24893131 10.1038/ncomms4996 PMC4046113

[77] Martín-Guerrero SM, Markovinovic A, Mórotz GM, Salam S, Noble W, Miller CCJ. Targeting ER-mitochondria signaling as a therapeutic target for frontotemporal dementia and related amyotrophic lateral sclerosis. Front Cell Dev Biol. 2022;10:915931. 35693938 10.3389/fcell.2022.915931 PMC9184680

[78] Obara CJ, Nixon-Abell J, Moore AS, Motion of VAPB molecules reveals ER-mitochondria contact site subdomains. Nature. 2024;626:169–76. 38267577 10.1038/s41586-023-06956-y PMC10830423

[79] Brown CN, Bayer KU. Studying CaMKII: Tools and standards. Cell Rep. 2024;43:113982. 38517893 10.1016/j.celrep.2024.113982 PMC11088445

[80] Yasuda R, Hayashi Y, Hell JW. CaMKII: a central molecular organizer of synaptic plasticity, learning and memory. Nat Rev Neurosci. 2022;23:666–82. 36056211 10.1038/s41583-022-00624-2

[81] Hou ST, Jiang SX, Aylsworth A, CaMKII phosphorylates collapsin response mediator protein 2 and modulates axonal damage during glutamate excitotoxicity. J Neurochem. 2009;111:870–81. 19735446 10.1111/j.1471-4159.2009.06375.x

[82] Moutal A, White KA, Chefdeville A, Dysregulation of CRMP2 post-translational modifications drive its pathological functions. Mol Neurobiol. 2019;56:6736–55. 30915713 10.1007/s12035-019-1568-4 PMC6728212

[83] Siwecka N, Rozpędek-Kamińska W, Wawrzynkiewicz A, Pytel D, Diehl JA, Majsterek I. The structure, activation and signaling of IRE1 and Its role in determining cell fate. Biomedicines. 2021;9:156. 33562589 10.3390/biomedicines9020156 PMC7914947

[84] Lee JK, Kim NJ. Recent Advances in the inhibition of p38 MAPK as a potential strategy for the treatment of Alzheimer’s disease. Molecules. 2017;22:1287. 28767069 10.3390/molecules22081287 PMC6152076

[85] Zhao Y, Kuca K, Wu W, Hypothesis: JNK signaling is a therapeutic target of neurodegenerative diseases. Alzheimers Dement. 2022;18:152–8. 34032377 10.1002/alz.12370

[86] Yue J, López JM. Understanding MAPK signaling pathways in apoptosis. Int J Mol Sci. 2020;21:2346. 32231094 10.3390/ijms21072346 PMC7177758

[87] Tortarolo M, Veglianese P, Calvaresi N, Persistent activation of p38 mitogen-activated protein kinase in a mouse model of familial amyotrophic lateral sclerosis correlates with disease progression. Mol Cell Neurosci. 2003;23:180–92. 12812752 10.1016/s1044-7431(03)00022-8

[88] Bendotti C, Atzori C, Piva R, Activated p38MAPK is a novel component of the intracellular inclusions found in human amyotrophic lateral sclerosis and mutant SOD1 transgenic mice. J Neuropathol Exp Neurol. 2004;63:113–9. 14989597 10.1093/jnen/63.2.113

[89] Holasek SS, Wengenack TM, Kandimalla KK, Activation of the stress-activated MAP kinase, p38, but not JNK in cortical motor neurons during early presymptomatic stages of amyotrophic lateral sclerosis in transgenic mice. Brain Res. 2005;1045:185–98. 15910777 10.1016/j.brainres.2005.03.037

[90] Veglianese P, Lo Coco D, Bao Cutrona M, Activation of the p38MAPK cascade is associated with upregulation of TNF alpha receptors in the spinal motor neurons of mouse models of familial ALS. Mol Cell Neurosci. 2006;31:218–31. 16219474 10.1016/j.mcn.2005.09.009

[91] Dewil M, dela Cruz VF, Van Den Bosch L, Robberecht W. Inhibition of p38 mitogen activated protein kinase activation and mutant SOD1(G93A)-induced motor neuron death. Neurobiol Dis. 2007;26:332–41. 17346981 10.1016/j.nbd.2006.12.023

[92] Sama RR, Fallini C, Gatto R, ALS-linked FUS exerts a gain of toxic function involving aberrant p38 MAPK activation. Sci Rep. 2017;7:115. 28273913 10.1038/s41598-017-00091-1 PMC5428330

[93] Kuijpers M, Yu KL, Teuling E, Akhmanova A, Jaarsma D, Hoogenraad CC. The ALS8 protein VAPB interacts with the ER-Golgi recycling protein YIF1A and regulates membrane delivery into dendrites. EMBO J. 2013;32:2056–72. 23736259 10.1038/emboj.2013.131 PMC3715857

[94] Tran D, Chalhoub A, Schooley A, Zhang W, Ngsee JK. A mutation in VAPB that causes amyotrophic lateral sclerosis also causes a nuclear envelope defect. J Cell Sci. 2012;125:2831–6. 22454507 10.1242/jcs.102111

[95] Zhao YG, Liu N, Miao G, Chen Y, Zhao H, Zhang H. The ER contact proteins VAPA/B interact with multiple autophagy proteins to modulate autophagosome biogenesis. Curr Biol. 2018;28:1234–1245.e4. 29628370 10.1016/j.cub.2018.03.002

[96] Karagas NE, Gupta R, Rastegari E, Loss of Activity-induced mitochondrial ATP production underlies the synaptic defects in a drosophila model of ALS. J Neurosci. 2022;42:8019–37. 36261266 10.1523/JNEUROSCI.2456-21.2022 PMC9617612

[97] Wong HC, Lang AE, Stein C, Drerup CM. ALS-linked VapB P56S mutation alters neuronal mitochondrial turnover at the synapse. J Neurosci. 2024:44. 10.1523/JNEUROSCI.0879-24.2024 PMC11358610 39054069

