# Supplementary material for: Pathogenic mechanisms of amyotrophic lateral sclerosis-linked VAPB P56S mutation in the degeneration of corticospinal motor neurons
Source: Ageing Neurodegener Dis. Author manuscript; Available in PMC 2026 Jan 14. (PMC12797193; doi:10.20517/and.2025.21)
Supplement: Supplementary Material [file NIHMS2134981-supplement-Supplementary_Material.pdf]

DOI: 10.20517/and.xxx.xx

Ageing and
Neurodegenerative Diseases

Supplementary Materials

Pathogenic mechanisms of amyotrophic lateral sclerosis-linked VAPB P56S mutation in the degeneration of corticospinal motor neurons

Xuan Yang[1,#], Jiayin Zheng[1,#], Xinyu Wang[1], Huaibin Cai[2], Jia Yu[1]

[1]Basic Research Center, Institute for Geriatrics and Rehabilitation, Beijing Geriatric Hospital, Beijing 100095, China.
[2]Transgenic Section, Laboratory of Neurogenetics, National Institute on Aging, National Institutes of Health, Bethesda, MD 20892, USA.
[#]The authors contributed equally to this work.

Correspondence to: Dr. Jia Yu, Basic Research Center, Institute for Geriatrics and Rehabilitation, Beijing Geriatric Hospital, 118 Wenquan Road, Haidian District, Beijing 100095, China. Email: jyu319@163.com; Dr. Huaibin Cai, Transgenics Section, Laboratory of Neurogenetics, National Institute on Aging, National Institutes of Health, Building 35, Room 1A112, MSC 3707, 35 Convent Drive, Bethesda, MD 20892, USA. Email: caih@mail.nih.gov

Supplementary Figure 1. Downregulated protein level and cytoplasmic inclusions of mutant VAPB in various types of neurons of P56S KI mice.
Supplementary Figure 2. Downregulated protein level and cytoplasmic inclusions of mutant VAPB in cultured cortical neurons of P56S KI mice.
Supplementary Figure 3. No apparent loss of hippocampal CA2 neurons, striatal neurons, cerebellar Purkinje neurons, and lumbar SMNs in aged P56S KI mice.

Supplementary Figure 4. VAPB P56S mutation disrupts ER-mitochondria contacts and calcium homeostasis.

© The Author(s) 2021. Open Access This article is licensed under a Creative Commons Attribution 4.0 International License (https://creativecommons.org/licenses/by/4.0/), which permits unrestricted use, sharing, adaptation, distribution and reproduction in any medium or format, for any purpose, even commercially, as long as you give appropriate credit to the original author(s) and the source, provide a link to the Creative Commons license, and indicate if changes were made.

Supplementary Figure 5. VAPB P56S mutation does not affect the PERK-eIF2α-ATF4-CHOP and ATF6 branches of the UPR signaling pathway.

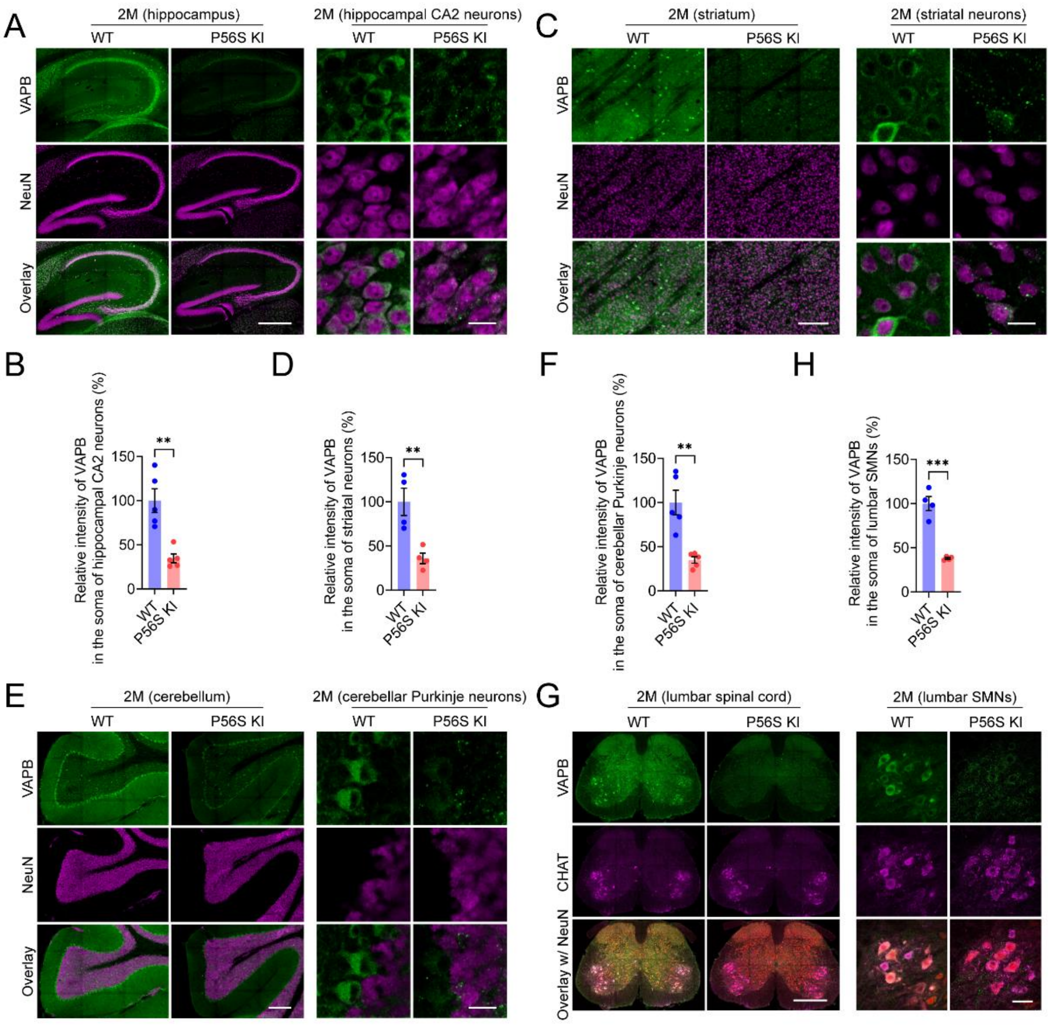

Supplementary Figure 1. Downregulated protein level and cytoplasmic inclusions of mutant VAPB in various types of neurons of P56S KI mice.

(A, B) Immunostaining of VAPB (green) and NeuN (purple) in the hippocampus of 2-month-old WT and P56S KI mice. Note the reduced intensity and cytoplasmic inclusions of mutant VAPB in the hippocampal CA2 neurons of P56S KI mice. Scale bar: 500 μm (low-magnification images), 20 μm (high-magnification images). The staining intensity of VAPB in the soma of hippocampal CA2 neurons was quantified as mean ± SEM (n = 5 animals per genotype and ≥20 neurons per animal). Unpaired *t* test, ** p = 0.0018.

(C, D) Immunostaining of VAPB (green) and NeuN (purple) in the striatum of 2-month-old WT and P56S KI mice. Note the reduced intensity and cytoplasmic inclusions of

mutant VAPB in the striatal neurons of P56S KI mice. Scale bar: 200 μm (low-magnification images), 20 μm (high-magnification images). The staining intensity of VAPB in the soma of striatal neurons was quantified as mean ± SEM (n = 4 animals per genotype and ≥20 neurons per animal). Unpaired t test, ** p = 0.0083.

(E, F) Immunostaining of VAPB (green) and NeuN (purple) in the cerebellum of 2-month-old WT and P56S KI mice. Note the reduced intensity and cytoplasmic inclusions of mutant VAPB in the cerebellar Purkinje neurons of P56S KI mice. Scale bar: 200 μm (low-magnification images), 20 μm (high-magnification images). The staining intensity of VAPB in the soma of cerebellar Purkinje neurons was quantified as mean ± SEM (n = 5 animals per genotype and ≥20 neurons per animal). Unpaired t test, ** p = 0.0019.

(G, H) Immunostaining of VAPB (green), NeuN (red), CHAT (purple) in the lumbar spinal cord of 2-month-old WT and P56S KI mice. SMNs located in the ventral horn were visualized by CHAT staining. Note the reduced intensity and cytoplasmic inclusions of mutant VAPB in the SMNs of P56S KI mice. Scale bar: 200 μm (low-magnification images), 20 μm (high-magnification images). The staining intensity of VAPB in the soma of SMNs was quantified as mean ± SEM (n = 4 animals per genotype and ≥20 neurons per animal). Unpaired t test, *** p = 0.0002.

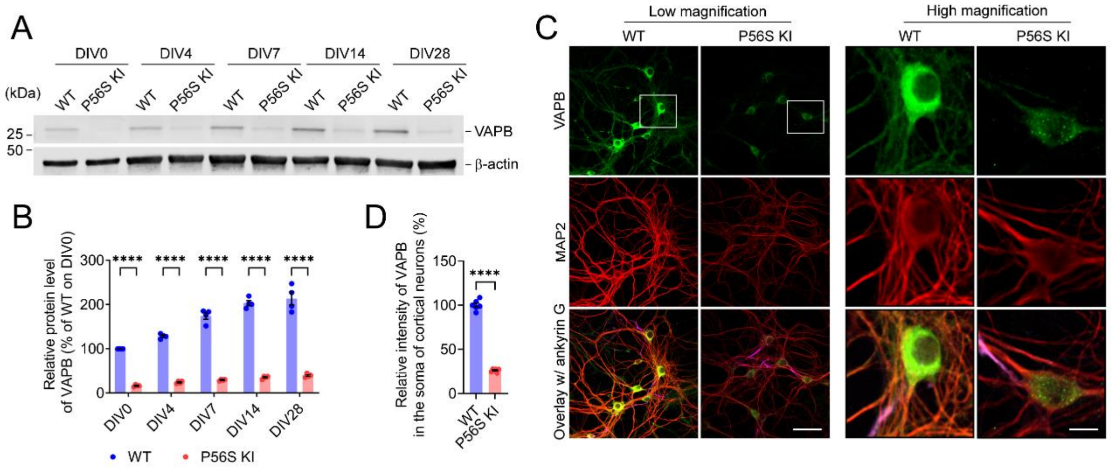

Supplementary Figure 2. Downregulated protein level and cytoplasmic inclusions of mutant VAPB in cultured cortical neurons of P56S KI mice. (A, B) Western blotting of VAPB in cultured cortical neurons from WT and P56S KI mice on 0, 7, 14, and 28 days in vitro (DIV). The protein level was quantified as mean ± SEM (at each time point, n = 4 independent cultures per genotype). Two-way ANOVA, **** p < 0.0001

(DIV0, DIV4, DIV7, DIV14, and DIV21). (C, D) Immunostaining of VAPB (green), MAP2 (red), and ankyrin G (purple) in DIV28 WT and P56S KI cortical neurons. The neuronal soma and dendrites were visualized by MAP2 staining. The axonal initial segment (AIS) was visualized by ankyrin G staining. Note the reduced intensity of mutant VAPB in the somata, dendrites, and axons of P56S KI neurons and the cytoplasmic inclusions of mutant VAPB. Scale bar: 50 µm (low magnification), 10 µm (high magnification). The staining intensity of VAPB in the soma of cortical neurons was quantified as mean ± SEM (n = 6 independent cultures per genotype and ≥20 neurons per culture). Unpaired *t* test, **** $p < 0.0001$.

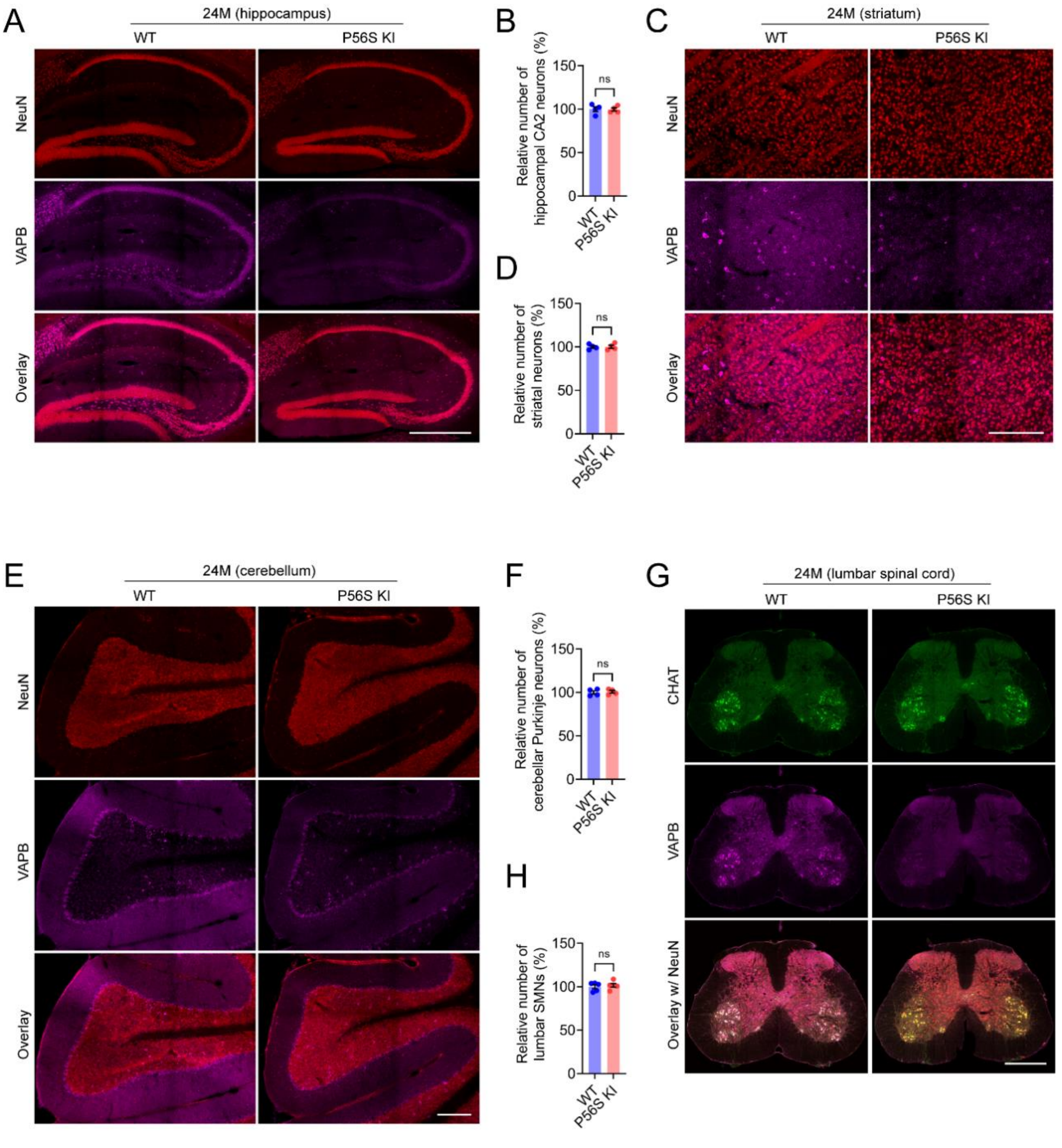

Supplementary Figure 3. No apparent loss of hippocampal CA2 neurons, striatal neurons, cerebellar Purkinje neurons, and lumbar SMNs in aged P56S KI mice.(A, B) Immunostaining of NeuN (red) and VAPB (purple) in the hippocampus of 24-month-old WT and P56S KI mice. Scale bar: 500 µm. The relative number of hippocampal CA2 neurons was quantified as mean ± SEM (n = 4 animals per genotype and 5 sections per animal). Unpaired *t* test, not significant (ns) p = 0.9376. (C, D) Immunostaining of NeuN (red) and VAPB (purple) in the striatum of 24-month-old WT and P56S KI mice. Scale bar: 200 µm. The relative number of striatal neurons was quantified as mean ± SEM (n = 4 animals per genotype and 5 sections per animal). Unpaired *t* test, ns p = 0.9483. (E, F) Immunostaining of NeuN (red) and VAPB (purple) in the cerebellum of 24-month-old WT and P56S KI mice. Scale bar: 200 µm. The relative number of cerebellar Purkinje neurons was quantified as mean ± SEM (n = 4 animals per genotype and 5 sections per animal). Unpaired *t* test, ns p = 0.7848. (G, H) Immunostaining of CHAT (green), NeuN (red), and VAPB (purple) in the lumbar spinal cord of 24-month-old WT and P56S KI mice. Unbiased stereology was performed to estimate the number of lumbar SMNs ($CHAT^+$ neurons located in the ventral horn) of 24-month-old WT and P56S KI mice. Scale bar: 500 µm. The relative number of lumbar SMNs was quantified as mean ± SEM (n = 5 animals). Unpaired *t* test, ns p = 0.6137.

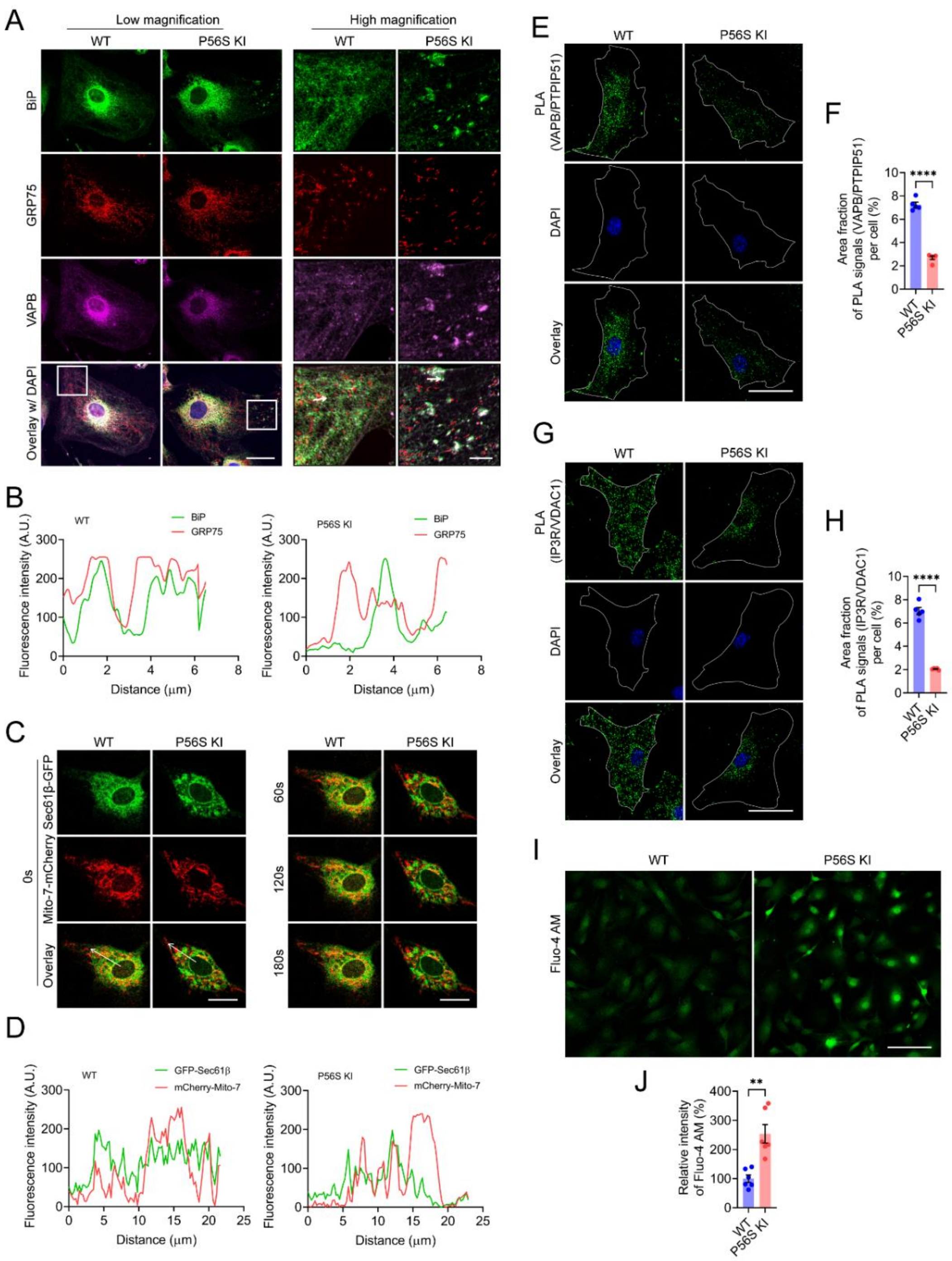

Supplementary Figure 4. VAPB P56S mutation disrupts ER-mitochondria contacts and calcium homeostasis. (A, B) Immunostaining of BiP (green), GRP75 (red), and VAPB (purple) in DIV14 WT and P56S KI fibroblasts. Scale bar: 40 μm (low magnification), 10 μm (high magnification). Line plots show the relative fluorescence intensity of each channel at the points along the arrow. Note the reduced ER-mitochondria contacts in P56S KI fibroblasts. (C, D) Live imaging of DIV14 WT and P56S KI fibroblasts co-expressing Sec61β-GFP (green) and Mito-7-mCherry (red).

Images at 0, 60, 120, and 180 seconds were presented. Scale bar: 20 μm. Line plots show the relative fluorescence intensity of each channel at the points along the arrow. Note the reduced ER-mitochondria contacts in P56S KI fibroblasts. (E, F) Proximity ligation assay (PLA) of VAPB-PTPIP51 interaction in DIV14 WT and P56S KI fibroblasts. Scale bar: 50 μm. Note the impaired VAPB-PTPIP51 interaction in P56S KI fibroblasts. The area fraction of PLA signals in the fibroblasts was quantified as mean ± SEM (n = 5 independent cultures per genotype and ≥20 fibroblasts per culture). Unpaired *t* test, **** $p < 0.0001$. (G, H) PLA of IP3R-VDAC1 interaction in DIV14 WT and P56S KI fibroblasts. Scale bar: 50 μm. Note the impaired IP3R-VDAC1 interaction in P56S KI fibroblasts. The area fraction of PLA signals in the fibroblasts was quantified as mean ± SEM (n = 5 independent cultures per genotype and ≥20 fibroblasts per culture). Unpaired *t* test, **** $p < 0.0001$. (I, J) Live imaging of DIV14 WT and P56S KI fibroblasts incubated with the cytosolic Ca^{2+} probe, Fluo-4 AM. Scale bar: 100 μm. The labeling intensity of Fluo-4 AM in the live fibroblasts was quantified as mean ± SEM (n = 6 independent cultures per genotype and ≥20 fibroblasts per culture). Unpaired *t* test, ** $p = 0.0012$.

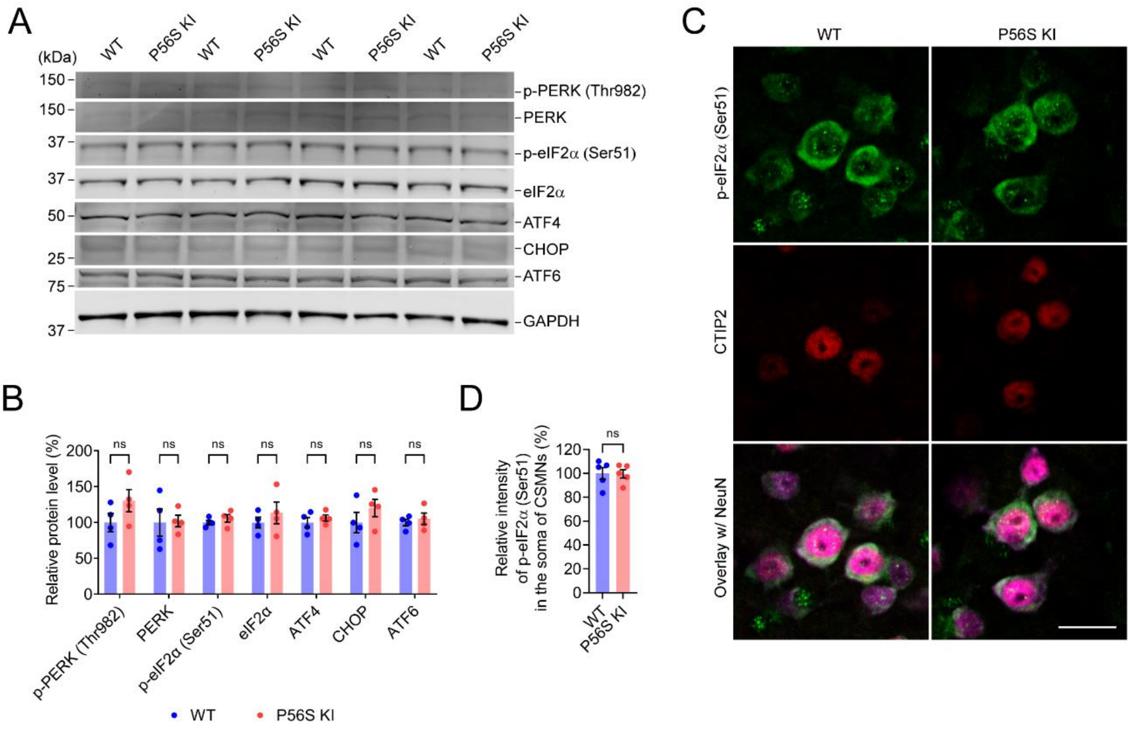

Supplementary Figure 5. VAPB P56S mutation does not affect the PERK-eIF2α-

ATF4-CHOP and ATF6 branches of the UPR signaling pathway. (A, B) Western blotting of p-PERK (Thr982), PERK, p-eIF2α (Ser51), eIF2α, ATF4, CHOP, and ATF6 in the cortex of 12-month-old WT and P56S KI mice. The protein level was quantified as mean ± SEM (n = 4 animals per genotype). Unpaired *t* test, not significant (ns) p = 0.0541 [p-PERK (Thr982)], ns p = 0.8897 (PERK), ns p = 0.7042 [p-eIF2α (Ser51)], ns p = 0.3829 (eIF2α), ns p = 0.6783 (ATF4), ns p = 0.1918 (CHOP), ns p = 0.7369 (ATF6). (C, D) Immunostaining of p-eIF2α (Ser51) (green), CTIP2 (red), and NeuN (purple) in the motor cortex layer V of 12-month-old WT and P56S KI mice. Scale bar: 20 μm. The staining intensity of p-eIF2α (Ser51) in the soma of CSMNs was quantified as mean ± SEM (n = 5 animals per genotype and ≥20 neurons per animal). Unpaired *t* test, ns p = 0.9415.
